# Comparison of methods for handling covariate missingness in propensity score estimation with a binary exposure

**DOI:** 10.1186/s12874-020-01053-4

**Published:** 2020-06-26

**Authors:** Donna L. Coffman, Jiangxiu Zhou, Xizhen Cai

**Affiliations:** 1 Temple University, 1301 Cecil B. Moore Ave. Ritter Annex, 9th floor, Philadelphia, PA 19122 USA; 2 GlaxoSmithKline, Philadelphia, USA; 3 Williams College, Williamstown, USA

**Keywords:** Propensity scores, Missing data, Causal inference, Generalized boosted models

## Abstract

**Background:**

Causal effect estimation with observational data is subject to bias due to confounding, which is often controlled for using propensity scores. One unresolved issue in propensity score estimation is how to handle missing values in covariates.

**Method:**

Several approaches have been proposed for handling covariate missingness, including multiple imputation (MI), multiple imputation with missingness pattern (MIMP), and treatment mean imputation. However, there are other potentially useful approaches that have not been evaluated, including single imputation (SI) + prediction error (PE), SI + PE + parameter uncertainty (PU), and Generalized Boosted Modeling (GBM), which is a nonparametric approach for estimating propensity scores in which missing values are automatically handled in the estimation using a surrogate split method. To evaluate the performance of these approaches, a simulation study was conducted.

**Results:**

Results suggested that SI + PE, SI + PE + PU, MI, and MIMP perform almost equally well and better than treatment mean imputation and GBM in terms of bias; however, MI and MIMP account for the additional uncertainty of imputing the missingness.

**Conclusions:**

Applying GBM to the incomplete data and relying on the surrogate split approach resulted in substantial bias. Imputation prior to implementing GBM is recommended.

## Background

Observational studies are common in epidemiology and medical studies when a randomized trial is not ethical or feasible. A major challenge facing observational studies is that the treatment or exposure is not randomized and the estimate of the effect of the exposure on an outcome may be due to confounders, variables associated with both the exposure and outcome. Propensity scores, the probability of receiving the observed exposure level given the covariates, are increasingly used to control for confounding. Propensity scores summarize many confounders into a single number [1] that can be used to reduce bias in the estimate of the effect of the exposure on an outcome. Using propensity scores to control for confounding assumes no unmeasured confounding and that all potential confounders are included in the propensity score model [1]. Thus, it is common to include many confounders in the propensity score model to satisfy the no unmeasured confounding assumption. However, there is often missingness in the confounders, which raises the question of how best to handle missingness when applying propensity score methods. There has been previous work around various imputation strategies (e.g., treatment mean imputation, multiple imputation [MI]) and how to combine results if multiple imputation is used. However, one approach that has not been examined in simulation studies when there is missingness on the confounders is Generalized Boosted Models (GBM) [2]. GBM does not require complete data (as the commonly used logistic regression does) and has been shown to outperform logistic regression for propensity score estimation in complete data [3]. If GBM works well in the presence of missing data, using it would circumvent the issues that arise when using MI with propensity score analysis [4–6]. The goal of this paper is to examine the performance of GBM vs. other approaches through a simulation study in order to provide guidance to analysts when implementing propensity score analysis in the presence of missingness on the covariates.

The paper is organized as follows. We briefly review the literature on missingness in the covariates when estimating propensity scores for binary exposures using logistic regression. We then briefly review previous work showing that GBM outperforms logistic regression, particularly in the presence of non-linear relationships, which leads to our choice of data generating scenarios for the simulation study. We then present the simulation study methods and the results in detail. We conclude with recommendations for researchers applying propensity score methods, specifically propensity score weighting, in the presence of missingness on the potential confounders.

### Methods for handling covariate missingness

Missingness on the propensity score model covariates poses a challenge in propensity score estimation. As propensity score estimation frequently involves a large number of covariates, a large proportion of observations may contain missingness on at least one covariate, and proper handling of covariate missingness is necessary for unbiased estimates of causal effects. Several approaches have been proposed for handling covariate missingness, including using only covariates with complete data, treatment mean imputation, [7] indicator variable, [8, 9] missingness pattern (MP), [10] the general location model, [11] MI, and multiple imputation missingness pattern (MIMP) [12]. Several of these approaches will not be considered further. Specifically, using only covariates with complete data is not recommended, [7] the indicator variable approach is not recommended [9] as it ignores any relation between covariates and is inefficient, [12] and the MP approach is less efficient than MI or the MIMP approach discussed below [7]. The general location model, [11] which models the joint distribution of the exposure, covariates, and missingness, is computationally intensive in practice. We review the remaining approaches in turn.

#### Treatment mean imputation

Treatment mean imputation has been suggested [7] as an alternative to MI when MI is not feasible because in a simulation study in which both the exposure and outcome were binary, treatment mean imputation performed reasonably well. Based on these results, we include the approach here in our simulation of a binary exposure and continuous outcome but speculate that if the missingness mechanism is anything other than missing completely at random (MCAR), this approach will not perform well.

#### Multiple imputation (MI)

A commonly used technique for handling missing data, MI could also be adopted for propensity score covariate missingness under the assumption of missing at random (MAR) [13]. Multiple random draws from the posterior predictive distribution of the missing values conditional on the observed values are used to generate multiple complete datasets. Each complete dataset can be separately analyzed with standard methods and estimates are combined using Rubin’s rules [14, 15]. The overall estimate ($\hat{\theta}\Big)$ is calculated as the average of point estimates (${\hat{\theta}}_i\Big)$ over the *m* imputations:

$$ \hat{\theta}=\frac{1}{m}\sum \limits_{i=1}^m{\hat{\theta}}_i $$

and the overall variance ($\hat{\mathit{\operatorname{var}}}\Big)$ is estimated as the sum of within-imputation variance (*W*_*i*_) and between-imputation variance (*B*):

$$ \hat{\mathit{\operatorname{var}}}=\frac{1}{m}{\sum}_{i=1}^m{W}_i+\left(1+\frac{1}{m}\right)B. $$

However, Rubin’s rules for combining variance estimates may not be valid when MI is applied to propensity score model covariates due to the extra uncertainty of estimating propensity scores. Instead, a bootstrap or jackknife method could be applied for variance estimation [12]. Using MI to handle missingness allows inclusion of auxiliary variables (i.e., variables associated with the missingness) in the imputation model. It has been suggested that both the exposure and outcome variables should be included in the imputation model to avoid biased estimates [7]. To implement MI, we used a fully conditional specification [16] using the MI via chained equations algorithm in the R package *mice* [17, 18].

Several issues arise when using MI in propensity score analysis, including whether the causal effects estimated on each imputed data set should be combined or whether the propensity score for each individual should be combined across imputations [4, 5] prior to estimating the causal effect. Although this latter approach has been recommended, [5] it is not clear that Rubin’s rules apply to this approach [4]. Other issues, which have not been addressed when using MI with propensity score analysis, include strategies for handling a situation in which balance is obtained in some imputations but not others.

#### Multiple imputation Missingness pattern (MIMP)

Qu and Lipkovich [12] introduced a new approach to handling covariate missingness by combining MI and MP. As missingness on the covariates may be predictive of treatment assignment, incorporating the MP in the propensity score model has been suggested [10]. Observations are classified into groups based on the MP and assigned an indicator. Missing values are imputed with MI and the propensity scores are estimated using the covariates and an MP indicator for each imputed dataset. The estimated propensity score is thus conditional on both observed covariates and the MP. Including the MP indicator in the propensity score model balances MPs across exposure groups. This is important because in order for the strong ignorability assumption to hold balance must be obtained for the missing as well as the observed values of the potential confounders [10, 19]. Finally, the causal effect is estimated for each imputed dataset and combined with Rubin’s rules (point estimate) and the bootstrap (variance).

One challenge with the MIMP approach is that with a large number of covariates, it is not uncommon to observe MPs containing very few observations, resulting in estimated propensity scores with large variability. This could be solved by pooling similar MPs to reach a pre-specified minimum number of observations [12].

#### Single imputation (SI)

As only a point estimate of the propensity score is used in the outcome analysis model, one could argue that a SI that incorporates prediction error (SI+ prediction error, or SI + PE) or a SI that incorporates both prediction error and parameter uncertainty (SI+ prediction error + parameter uncertainty, or SI + PE + PU) may be sufficient to eliminate bias due to missingness in the propensity score model covariates. However, one could also argue that the uncertainty of the missing data imputation should be carried over to the outcome analysis. Performance of SI + PE and SI + PE + PU has not been evaluated or compared with the methods discussed above even though they have been used in applications of propensity score analysis [20]. We suspect that either of these approaches would perform better than treatment mean imputation and would avoid the potential issues with MI described above. Thus, we consider two SI methods: SI + PE and SI + PE + PU.

SI + PE involves fitting a regression model of the variable containing missing values on the auxiliary variables and imputing the missing values by adding random noise to the predicted value. Although the approach may result in incorrect standard errors due to its inability to account for uncertainty in the regression coefficients, and therefore underestimate variability of the imputed values, [15] this may be less of an issue for propensity score estimation where only the point estimate of the propensity scores is used for later outcome analysis. SI + PE + PU is similar but uses Bayesian methods to draw the parameters from their posterior distributions.

### Propensity score estimation

#### Logistic regression

Logistic regression is most commonly used for propensity score estimation by regressing the binary treatment or exposure indicator variable on pre-treatment covariates (i.e., potential confounders). The propensity score model for a binary exposure variable is estimated as

$$ logit\left({p}_i\left(T=1\right)\right)=\boldsymbol{X}^{\prime}\boldsymbol{\beta}, \kern0.5em i=1,2,\dots, n, $$

where ***X*** = (1, *X*_1_, *X*_2_, …, *X*_*k*_), ***β*** = (*β*_0_, *β*_1_, *β*_2_, …, *β*_*k*_), *k* is the number of covariates, and *n* is the number of observations. The propensity score for each individual is then estimated as

$$ {p}_i=\frac{{\mathrm{e}}^{{\boldsymbol{X}}_i^{\prime}\boldsymbol{\beta}}}{1+{\mathrm{e}}^{{\boldsymbol{X}}_i^{\prime}\boldsymbol{\beta}}}. $$

Although logistic regression is most commonly used to estimate propensity scores, it may not be the best choice in some situations. Using logistic regression to model the probability of exposure level assumes a linear relationship between the log-odds of the exposure and the covariates. However, this assumption is not always satisfied and including lower- (e.g., the square root) or higher-order terms of covariates in the logistic model is unlikely to accurately capture nonlinear relationships [2]. Also, the propensity score model usually involves a large number of covariates and variable selection is often required. This process could be tedious and may exclude important covariates. In addition, inclusion of two-way or higher-order interaction terms in a logistic regression requires users to manually specify all interaction terms.

#### Generalized boosted modeling (GBM)

The limitations of estimating propensity scores using a parametric model, such as logistic regression, motivated development of more powerful and flexible nonparametric methods, including GBM [2]. GBM estimates propensity scores by iteratively fitting many regression trees using the covariates and linearly combines all regression trees to form a smoothed function for the final estimate of the propensity scores. Similar to logistic regression, GBM also models the log-odds of exposure level, $g(X)=\log \frac{p\left(T=1|\boldsymbol{X}\right)}{1-p\left(T=1|\boldsymbol{X}\right)}$. The algorithm starts with the average log-odds of exposure level, $g(X)=\log \frac{\hat{p}\left(T=1\right)}{1-\hat{p}\left(T=1\right)}$, where $\hat{p}\left(T=1\right)$ is the average probability of being exposed, and *g*(*X*) is iteratively updated to *g*(*X*) + *α* ∙ *h*(*X*) to find the model that maximizes the log-likelihood of *g*(*X*),

$$ l(g)={\sum}_{i=1}^n{T}_ig\left({X}_i\right)-\log \Big(1+\exp \left(g\left({X}_i\right)\right), $$

where *h*(*X*) is estimated by fitting a regression tree that models the residuals from the current fit to the covariates, and *α* (0 < *α* ≤ 1) is a shrinkage parameter which allows a smaller adjustment between iterations to reduce variance without necessarily increasing bias. A small shrinkage parameter helps achieve better model fit. Another tuning parameter in GBM is the interaction depth. A maximum depth of four, which allows all four-way interactions between covariates, is recommended for best model estimation and prediction [2]. The process continues until the maximum number of iterations is reached; propensity scores are calculated from the iteration where the average absolute standardized mean (AASM) difference is minimized. AASM is estimated as the average of absolute differences between the exposed mean and weighted unexposed mean across the *k* covariates:

$$ \frac{1}{k}\sum \limits_k\left|\frac{\sum_{i=1}^{n_{T=1}}{X}_k{\left(T=1\right)}_i-\frac{\sum_{j=1}^{n_{T=0}}{w}_j{X}_k{\left(T=0\right)}_j}{\sum_{j=1}^{n_{T=0}}{w}_j}}{\hat{sd}{\left({X}_k\right)}_{T=1}}\right| $$

where *w*_*j*_ = $$ \frac{p_j}{1-{p}_j} $$.

Using GBM for propensity score estimation has several advantages. First, GBM can adaptively include the covariates that improve prediction of exposure level, and therefore does not require covariate selection. Second, GBM automatically includes all higher-order and interaction terms of the covariates to the depth specified by the user. Thus, nonlinear relationships can be accounted for in propensity score estimation to yield more accurate estimates. Third, the iterative fitting nature of GBM can identify the propensity score model that minimizes the differences between exposure groups on the covariates. One disadvantage in comparison to logistic regression is that GBM does not provide parameter estimates, such as logistic regression coefficients, that allow interpretation of the association between a covariate and the probability of exposure. However, this is less of a concern as the main purpose of the propensity score model is to estimate the conditional probability of exposure.

One potentially important advantage of GBM over logistic regression for propensity score estimation is that GBM does not require complete data on the covariates. Missing values are handled using the surrogate split method. At each iteration, a regression tree, called a base learner, is built by splitting observations into left and right nodes. Suppose that a splitting variable and split point (for partitioning to the left and right nodes) have been chosen for the next split. This variable will be referred to as the primary variable. If there are missing values on the primary variable, these observations are sent to a separate third node, called the missing node. For this node, a surrogate variable is found by predicting the left and right nodes of the primary variable using the other predictor variables. If the values on both the primary variable and surrogate variable are missing, then a second-best surrogate split can be found that yields a splitting of the data most similar to the primary split. A simple rule of “go with the majority” is also evaluated, and surrogates that perform worse than this rule are not used for the surrogate split. Ultimately, the goal of the surrogate is to split the data as similarly as possible to the primary variable, although there is no guarantee that it does so.

Propensity scores estimates, whether estimated by GBM or logistic regression, can then be used to match, stratify, or weight observations to balance the covariate distributions across exposure groups. In this study, we will focus on inverse probability weighting (IPW). To reduce the variance that may be incurred by extreme weights, the weight is multiplied by the expected value of being assigned to the observed group [21]. Thus, the exposed group is weighted by $\frac{\hat{p}}{p_i}$, and the unexposed group is weighted by $\frac{1-\hat{p}}{1-{p}_i}$, where $\hat{p}=\frac{\sum_i^n{p}_i}{n}$.

## Methods

Although many methods have been proposed for handling covariate missingness and several have been shown to have good performance, SI + PE and SI + PE + PU have not been previously examined. Performance of GBM with incomplete covariates using the surrogate split has also not been evaluated in simulation studies, particularly in comparison to the other approaches described above. Cham and West examined the surrogate split approach with GBM in a single simulated data set but noted that more extensive simulation studies were needed [19]. To address these knowledge gaps, a simulation study was conducted.

### Data generation

**Fig. 1:**
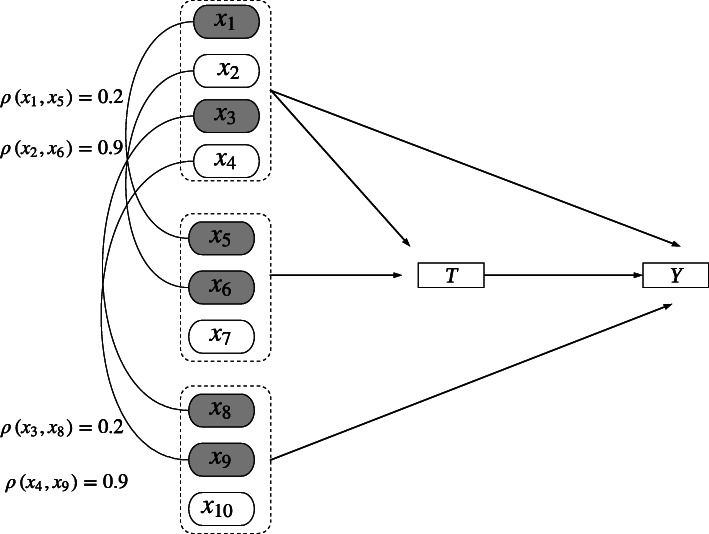
Complete data generation model

The data generation model is illustrated in Fig. 1. Complete data generation was similar to that of Setoguchi et al. [22] and Lee et al. [3] and included a binary exposure, denoted as *T*, a continuous outcome, denoted as *Y*, and 10 covariates, denoted as *x*_1_ − *x*_10_.

#### Covariates

Six covariates were binary (*x*_1_, *x*_3_, *x*_5_, *x*_6_, *x*_8_, *x*_9_) following a Bernoulli distribution with *x*_1_, *x*_6_, *x*_8_~*Bern* (1, 0.3) and *x*_3_, *x*_5_, *x*_9_~*Bern* (1, 0.5). Four covariates were continuous (*x*_2_, *x*_4_, *x*_7_, *x*_10_) following a normal distribution, *N*(0, 1). Four covariates (*x*_1_ − *x*_4_) were correlated with both *T* and *Y* and were true confounders. Three covariates (*x*_5_ − *x*_7_) were correlated only with *T* and the other three covariates (*x*_8_ − *x*_10_) were correlated only with *Y*. Covariate *x*_1_ was correlated with *x*_5_ (*ρ* = 0.2), *x*_2_ was correlated with *x*_6_ (*ρ* = 0.9), *x*_3_ was correlated with *x*_7_ (*ρ* = 0.2), and *x*_4_ was correlated with *x*_8_ (*ρ* = 0.9).

#### Exposure

Generation of the exposure was similar to Setoguchi et al. [22] and Lee et al.’s [3] scenarios A and G with sample sizes *n*= 500, 1000, and 5000. In scenario A (main effects only), linearity and additivity hold, so higher order terms and interactions among the confounders are not needed for the propensity score model. The true propensity model is given as:

$$ logit\left(p\left(T=1\right)\right)=0.8{x}_1-0.25{x}_2+0.6{x}_3-0.4{x}_4-0.8{x}_5-0.5{x}_6+0.7{x}_7. $$

In scenario G (moderate non-additivity and non-linearity), neither linearity nor additivity hold, so both higher order terms and interactions among the confounders should be included in the propensity score model (10 two-way interactions and 3 quadratic terms). The true propensity score model is given as:

$$ logit\left(p\left(T=1\right)\right)=0.8{x}_1-0.25{x}_2+0.6{x}_3-0.4{x}_4-0.8{x}_5-0.5{x}_6+0.7{x}_7-0.25{x}_2^2-0.4{x}_4^2+0.7{x}_7^2+0.4{x}_1{x}_3-0.175{x}_2{x}_4+0.3{x}_3{x}_5-0.28{x}_4{x}_6-0.4{x}_5{x}_7+0.4\ {x}_1{x}_6-0.175\ {x}_2{x}_3+0.3{x}_3{x}_4-0.2{x}_4{x}_5-0.4{x}_5{x}_6 $$

#### Outcome

The regression coefficient of *Y* on *T,* which is the true causal effect, was set to − 0.4. The true outcome model is given as:

$$ \kern0.5em Y=-3.85+0.3{x}_1-0.36{x}_2-0.73{x}_3-0.2{x}_4+0.71{x}_8-0.19{x}_9+0.26{x}_{10}-0.4T+{\varepsilon}_Y $$

where *ε*_*Y*_~*N*(0, 1).

### Missingness mechanisms

The design for the missingness mechanisms included four conditions where for each condition the overall rate of missingness was either 25% or 50%. The rationale for an overall rate of 50% missingness was that of a planned missingness design in which some individuals may receive subsets of questionnaire items [23]. The missingness mechanisms were based on the simulation design of Collins, Schafer, and Kam [24] but adapted, as described below, to our design for studying covariate missingness in the propensity score model. The rationale for the design of the missingness mechanisms is that in real empirical data, the missingness mechanism likely does not fall into discrete categories of MCAR, MAR, and MNAR. We do not include a condition in which the missingness is MNAR because all of the approaches we examine assume that the missingness is not MNAR and thus, all of the approaches would be expected to be biased under MNAR. We do, however, include different MAR conditions that are likely in real data.

*MCAR*. For MCAR, approximately 25% or 50% of observations were missing randomly (i.e., independently of any other covariate, the exposure, or the outcome) for each of the four true confounders, *x*_1_ − *x*_4_.

*MAR-1*. For MAR 1, missingness on *x*_1_, *x*_2_, *x*_3_, *x*_4_ is dependent on *x*_5_, *x*_6_, *x*_8_, *x*_9_, respectively. Specifically, approximately 15% or 30% of observations are missing on *x*_1_ when *x*_5_ is equal to 0, and approximately 35% or 70% of observations are missing on *x*_1_ when *x*_5_ is equal to 1, yielding overall missingness percentages of 25% or 50%; *x*_2_, *x*_3_, *x*_4_ follow the same pattern. Note that although *x*_5_ − *x*_7_ predict only the exposure, *x*_5_ and *x*_6_ are correlated with the true confounders (*x*_1_ and *x*_2_ respectively; see Fig. 1). Similarly, although *x*_8_ − *x*_10_ predict only the outcome, *x*_8_ and *x*_9_ are correlated with the true confounders (*x*_3_ and *x*_4_ respectively). Thus, these variables (*x*_5_, *x*_6_, *x*_8_, *x*_9_) are important auxiliary variables that should be included in the imputation model in order to satisfy the MAR assumption. If these variables are not included, then this mechanism is missing not at random (MNAR).

*MAR-2*. For MAR 2, missingness on *x*_1_, *x*_2_, *x*_3_, *x*_4_ is dependent on *x*_5_, *x*_6_, *x*_8_, *x*_9_, respectively, *and Y*. To generate missingness on *x*_1_, all observations were first divided into four groups based on the values of *x*_5_ and *Y* (see Table 1). Each group was then assigned a specific missingness percentage and missing values were introduced within each group, yielding an overall missingness percentage of 25% or 50%. Missingness was generated on covariates *x*_2_, *x*_3_, *x*_4_ using group specific missing percentages as shown in Tables 2, 3, and 4. For this condition, *x*_5_, *x*_6_, *x*_8_, *x*_9_, and *Y* must be included in the imputation model in order to satisfy the MAR assumption. If these variables are not included, then this mechanism is MNAR.

**Table 1:** Percentages of missingness on *x*_1_ based on *x*_5_ and *Y*

Group	*x*_5_	*I*{*Y* > *mean*(*Y*)}	% of observations	% missing on *x*_1_	% missing on *x*_1_
1	0	0	25	10	20
2	0	1	25	20	40
3	1	0	25	30	60
4	1	1	25	40	80
Total				25	50

**Table 2:** Percentages of missingness on *x*_2_ based on *x*_6_ and *Y*

Group	*x*_6_	*I*{*Y* > *mean*(*Y*)}	% of observations	% missing on *x*_2_	% missing on *x*_2_
1	0	0	30	25	50
2	0	1	40	30	60
3	1	0	20	20	40
4	1	1	10	15	30
Total				25	50

**Table 3:** Percentages of missingness on *x*_3_ based on *x*_8_ and *Y*

Group	*x*_8_	*I*{*Y* > *mean*(*Y*)}	% of observations	% missing on *x*_3_	% missing on *x*_3_
1	0	0	40	30	60
2	0	1	30	25	50
3	1	0	10	15	30
4	1	1	20	20	40
Total				25	50

**Table 4:** Percentages of missingness on *x*_4_ based on *x*_9_ and *Y*

Group	*x*_9_	*I*{*Y* > *mean*(*Y*)}	% of observations	% missing on *x*_4_	% missing on *x*_4_
1	0	0	20	15	30
2	0	1	30	25	50
3	1	0	30	35	70
4	1	1	20	20	40
Total				25	50

*MAR-sinister*. For MAR sinister, missingness on *x*_1_, *x*_2_, *x*_3_, *x*_4_ is dependent on the correlation of two other variables. Specifically, missingness on *x*_1_ is dependent on the correlation between *x*_5_ and *T*. The observations were first randomly divided into 20 groups and the correlation between *x*_5_ and *T* was calculated for each group. The 10 groups with lower correlation were assigned a missingness percentage of either 10% or 30% and the 10 groups with higher correlation were assigned a missingness percentage of either 40% or 70%, resulting in an overall missingness percentage of 25% or 50%. Missingness on *x*_2_, *x*_3_, *x*_4_ was generated based on the correlation between *x*_6_ and *T*, *x*_7_ and *Y*, and *x*_8_ and *Y*, respectively. For this condition, *x*_5_, *x*_6_, *x*_8_, *x*_9_, *T,* and *Y* must be included in the imputation model in order to satisfy the MAR assumption. If these variables are not included, then this mechanism is MNAR.

### Analysis

Analysis began with imputation of missing data, followed by estimation of propensity scores, and finally estimation of the causal effect.

#### Imputation

Methods included treatment mean imputation, SI + PE, SI + PE + PU, MI, and MIMP using the R package *mice*. The imputation models include all variables except *x*_7_ and *x*_10_, which are not correlated with *x*_1_, *x*_2_, *x*_3_, *x*_4_. To implement the SI + PE method, we used the *mice* function from the R *mice* package with ‘method = norm.nob’ [18]. To implement the SI + PE + PU method, we used the *mice* function with ‘method = norm’ and *m* = 1 [18]. For MI and MIMP, we used *m =* 20 imputations. For treatment mean imputation, missing values in binary variables *x*_1_ and *x*_3_ are replaced by the unrounded mean of observed values within each exposure group, as rounding has been suggested to increase bias [25]. For MIMP, missingness patterns with less than 100 observations were pooled using the algorithm proposed by Qu and Lipkovich [12].

#### Propensity score estimation

To estimate propensity scores, we used logistic regression applied to the data imputed by treatment mean imputation, SI + PE, SI + PE + PU, MI, and MIMP; and GBM applied to the incomplete data and the data imputed by SI + PE. In practice, an analyst would not know the true propensity score model and would need to make decisions regarding which variables, as well as higher order terms and interactions, to include in the propensity score model. To examine how these decisions may affect the causal effect estimate, we considered four variable inclusion strategies for the logistic regression propensity score model:

a) true confounders *x*_1_, *x*_2_, *x*_3_, *x*_4_,

b) *x*_1_ left out,

c) nonconfounder *x*_5_ included and,

d) *x*_1_ left out and *x*_5_ included.

When logistic regression was used for propensity score estimation, only main effects were considered and no higher order terms or interactions were included in the model. As GBM is computationally time consuming, it was only implemented for strategy a) true confounders, using the *ps* function in the R package *twang* [26]. The shrinkage parameter was set to 0.05*.*

#### Estimation of the causal effect

To estimate the causal effect, we used a weighted regression model in which the IPWs were the weights and the exposure variable was the only predictor of the outcome (i.e., we did not incorporate the confounders in the outcome analysis model). Weights were trimmed at the 1st and 99th percentiles of their distribution. For MI and MIMP, estimates were combined using Rubin’s rules by taking the average of the estimates over the *m* = 20 imputations.

#### Evaluation

To summarize, there are 8 data generating scenarios: 2 (exposure models) × 4 (missingness mechanisms); 7 propensity score estimation strategies; and 4 variable inclusion strategies. The procedure was replicated 1000 times in each of these conditions before summarizing results for the different approaches. Approaches are compared in terms of i) bias, calculated as the difference between the mean estimate across the 1000 replications in each condition and the true value; ii) standard deviation (SD) of the estimates from the 1000 replications, iii) standard error (SE), estimated from the weighted regression in each replication and averaged across the 1000 replications, and iv) root mean square error (RMSE), calculated as the mean square of the differences between each estimate (${\hat{\beta}}_i\Big)$ and the true value (*β*):

$$ RMSE=\sqrt{\frac{1}{r}\sum \limits_{i=1}^r{\left({\hat{\beta}}_i-\beta \right)}^2} $$

## Results

### Scenario a – Main effects only

Table 5 shows simulation results for scenario A with *n*= 500 and 25% missingness. When only true confounders were used as covariates for propensity score estimation, there was no bias associated with SI + PE, SI + PE + PU, MI, and MIMP under all missingness mechanisms, and the bias was comparable to that obtained prior to introducing missing data (see first row of Table 5). Logistic regression with treatment mean imputation had a larger bias than other imputation approaches under all missingness mechanisms. Although not as large as treatment mean imputation, the bias obtained when applying GBM to incomplete data for propensity score estimation was substantially more than the SI or MI approaches. Imputing missing values by SI + PE before estimating propensity scores using GBM helped reduce the bias, but it was still larger than the bias obtained when applying GBM to complete data prior to introducing missingness (see second row of Table 5). GBM using incomplete data had slightly smaller SEs than the other approaches, whereas GBM with SI + PE had SEs in line with the other SI and MI approaches and with the analysis on the complete data prior to introducing missingness. Treatment mean imputation had larger SEs than the other approaches.

**Table 5:** Simulation results for scenario A, n = 500, 25%missing

Missingness Mechanism	Method	True confounders			Leave ×1 out			Add ×5			Leave ×1 out + Add ×5		
		Bias (SD)	SE	RMSE	Bias (SD)	SE	RMSE	Bias (SD)	SE	RMSE	Bias (SD)	SE	RMSE
	complete	−0.001 (0.041)	0.068	0.041	0.036 (0.043)	0.067	0.056	−0.001 (0.043)	0.070	0.043	0.042 (0.043)	0.068	0.060
	comGBM	0.029 (0.042)	0.067	0.050	–	–	–	–	–	–	–	–	–
**MCAR**	SI + pe + pu	0.000 (0.049)	0.068	0.041	0.036 (0.049)	0.067	0.061	0.000 (0.051)	0.070	0.051	0.042 (0.050)	0.068	0.065
	SI + pe	−0.003 (0.049)	0.068	0.041	0.035 (0.048)	0.067	0.059	−0.002 (0.050)	0.070	0.050	0.041 (0.049)	0.068	0.064
	TMI	0.105 (0.065)	0.072	0.113	0.131 (0.064)	0.071	0.146	0.109 (0.066)	0.074	0.127	0.138 (0.066)	0.072	0.153
	MI	−0.001 (0.045)	0.068	0.041	0.036 (0.046)	0.067	0.058	−0.001 (0.047)	0.070	0.047	0.042 (0.047)	0.068	0.063
	MIMP	−0.001 (0.045)	0.069	0.041	0.035 (0.046)	0.067	0.058	−0.001 (0.047)	0.070	0.047	0.042 (0.047)	0.068	0.063
	GBM	0.067 (0.050)	0.066	0.079	–	–	–	–	–	–	–	–	–
	GBM + SI + pe	0.035 (0.047)	0.067	0.054	–	–	–	–	–	–	–	–	–
**MAR1**	SI + pe + pu	0.000 (0.048)	0.068	0.041	0.036 (0.048)	0.067	0.060	0.000 (0.050)	0.070	0.050	0.042 (0.049)	0.068	0.065
	SI + pe	0.000 (0.048)	0.068	0.041	0.036 (0.047)	0.067	0.059	0.000 (0.050)	0.070	0.050	0.042 (0.048)	0.068	0.064
	TMI	0.102 (0.060)	0.070	0.110	0.129 (0.060)	0.069	0.142	0.109 (0.062)	0.071	0.125	0.136 (0.062)	0.070	0.149
	MI	0.000 (0.044)	0.068	0.041	0.036 (0.045)	0.067	0.058	0.000 (0.046)	0.070	0.046	0.042 (0.046)	0.068	0.062
	MIMP	0.000 (0.045)	0.069	0.041	0.037 (0.045)	0.067	0.058	0.000 (0.046)	0.070	0.046	0.043 (0.046)	0.068	0.063
	GBM	0.066 (0.048)	0.066	0.078	–	–	–	–	–	–	–	–	–
	GBM + SI + pe	0.036 (0.046)	0.067	0.055	–	–	–	–	–	–	–	–	–
**MAR2**	SI + pe + pu	0.000 (0.049)	0.068	0.041	0.036 (0.049)	0.067	0.061	0.001 (0.051)	0.070	0.051	0.043 (0.049)	0.068	0.065
	SI + pe	−0.001 (0.048)	0.068	0.041	0.035 (0.048)	0.067	0.059	−0.001 (0.050)	0.070	0.050	0.042 (0.049)	0.068	0.065
	TMI	0.104 (0.068)	0.073	0.112	0.129 (0.068)	0.072	0.146	0.111 (0.071)	0.075	0.132	0.137 (0.069)	0.073	0.153
	MI	0.000 (0.045)	0.068	0.041	0.037 (0.046)	0.067	0.059	0.001 (0.047)	0.070	0.047	0.043 (0.047)	0.068	0.064
	MIMP	0.002 (0.045)	0.069	0.041	0.038 (0.045)	0.067	0.059	0.001 (0.047)	0.070	0.047	0.043 (0.046)	0.068	0.063
	GBM	0.069 (0.050)	0.067	0.080	–	–	–	–	–	–	–	–	–
	GBM + SI + pe	0.037 (0.046)	0.067	0.055	–	–	–	–	–	–	–	–	–
**MAR Sinister**	SI + pe + pu	0.003 (0.048)	0.068	0.041	0.037 (0.048)	0.066	0.061	0.004 (0.049)	0.069	0.049	0.043 (0.048)	0.067	0.064
	SI + pe	−0.002 (0.049)	0.068	0.041	0.035 (0.049)	0.067	0.060	−0.002 (0.050)	0.070	0.050	0.041 (0.050)	0.068	0.065
	TMI	0.118 (0.062)	0.071	0.125	0.144 (0.062)	0.070	0.157	0.122 (0.064)	0.073	0.138	0.151 (0.064)	0.071	0.164
	MI	0.003 (0.045)	0.068	0.041	0.040 (0.045)	0.067	0.060	0.003 (0.046)	0.070	0.046	0.046 (0.046)	0.068	0.065
	MIMP	0.003 (0.045)	0.068	0.041	0.040 (0.046)	0.067	0.061	0.003 (0.046)	0.070	0.046	0.046 (0.046)	0.068	0.065
	GBM	0.074 (0.049)	0.066	0.085	–	–	–	–	–	–	–	–	–
	GBM + SI + pe	0.036 (0.047)	0.067	0.055	–	–	–	–	–	–	–	–	–

*Note.* Complete: logistic regression with complete data before introducing missingness; *comGBM* GBM with complete data before introducing missingness; *SI + pe + pu* single imputation + prediction error + parameter uncertainty; *SI + pe* single imputation + prediction error; *TMI* treatment mean imputation; *MI* multiple imputation (*m* = 20); *MIMP* multiple imputation missingness pattern (*m* = 20); *GBM* GBM with incomplete data; *GBM + SI + pe* GBM after single imputation + prediction error; *SD* standard deviation; *SE* standard error; *RMSE* root mean squared error

The causal effect estimates were affected by the variable inclusion strategies (see Table 5). When true confounder *x*_1_ was omitted from the propensity score model, an increase in bias was observed for all approaches under all missingness mechanisms; however, it was comparable to the bias obtained in the complete data prior to introducing missingness, with the exception of treatment mean imputation, which had much larger bias. The SEs did not differ in comparison to inclusion of only true confounders. When non-confounder *x*_5_ was included in the propensity score estimation, there was no increase in bias for the SI and MI approaches under all missingness mechanisms but there was increased bias for treatment mean imputation. The SEs slightly increased for all approaches in comparison to inclusion of only true confounders. When true confounder *x*_1_ was omitted and non-confounder *x*_5_ was included in the propensity score model, an even larger bias was observed for all approaches and all missingness mechanisms than when only true confounder *x*_1_ was omitted although the SEs did not increase. However, as before the SI and MI approaches have bias that is similar to that obtained in the complete data prior to introducing missingness.

The same pattern of results was obtained with sample sizes of 1000 or 5000 (except that as expected the SEs decreased) and thus the results are not presented here but are available in the supplementary materials. When the missingness increased from 25 to 50%, the bias for GBM using incomplete data increased quite substantially and GBM with SI + PE had slightly larger bias (see supplementary materials). For the SI and MI approaches using logistic regression, the bias increased for the MAR sinister mechanism and 50% missingness, that is, as the missingness mechanism became more complex. The increase to 50% missingness did not have an effect on SEs in comparison to 25% missingness but within the 50% missingness condition, the SEs decreased as the sample size increased. Treatment mean imputation performed at least as poorly in the 50% missingness condition as it did in the 25% missingness condition.

### Scenario G – moderate non-additivity and non-linearity

Table 6 shows simulation results for scenario G with *n*= 500 and 25% missingness. Recall that the approaches using logistic regression included only main effects. Thus, bias was observed for logistic regression and GBM when applied to the complete data prior to introducing missingness; however, for GBM, the bias was the same magnitude as in Scenario A.

**Table 6:** Simulation results for scenario G, *n* = 500, 25%missing

Missingness Mechanism	Method	True confounders			Leave ×1 out			Add ×5			Leave ×1 out + Add ×5		
		Bias (SD)	SE	RMSE	Bias (SD)	SE	RMSE	Bias (SD)	SE	RMSE	Bias (SD)	SE	RMSE
	complete	−0.014 (0.044)	0.071	0.046	0.037 (0.043)	0.068	0.057	−0.016 (0.045)	0.073	0.048	0.040 (0.043)	0.069	0.059
	comGBM	0.029 (0.044)	0.067	0.053	–	–	–	–	–	–	–	–	–
**MCAR**	SI + pe + pu	−0.011 (0.052)	0.071	0.053	0.040 (0.049)	0.068	0.063	−0.012 (0.054)	0.072	0.055	0.043 (0.050)	0.068	0.066
	SI + pe	−0.013 (0.051)	0.071	0.053	0.039 (0.049)	0.068	0.063	−0.014 (0.053)	0.072	0.055	0.042 (0.049)	0.068	0.065
	TMI	0.096 (0.064)	0.076	0.115	0.131 (0.061)	0.073	0.145	0.096 (0.067)	0.078	0.117	0.134 (0.063)	0.074	0.148
	MI	−0.011 (0.048)	0.071	0.049	0.039 (0.046)	0.068	0.060	−0.013 (0.050)	0.073	0.052	0.042 (0.047)	0.069	0.063
	MIMP	−0.011 (0.048)	0.072	0.049	0.039 (0.046)	0.068	0.060	−0.013 (0.050)	0.073	0.052	0.042 (0.047)	0.069	0.063
	GBM	0.060 (0.051)	0.067	0.079	–	–	–	–	–	–	–	–	–
	GBM + SI + pe	0.033 (0.049)	0.067	0.059	–	–	–	–	–	–	–	–	–
**MAR1**	SI + pe + pu	0.000 (0.051)	0.070	0.051	0.048 (0.048)	0.067	0.068	−0.002 (0.053)	0.072	0.053	0.052 (0.049)	0.068	0.071
	SI + pe	−0.001 (0.050)	0.070	0.050	0.048 (0.047)	0.067	0.067	−0.003 (0.052)	0.072	0.052	0.051 (0.048)	0.068	0.070
	TMI	0.113 (0.057)	0.073	0.127	0.148 (0.055)	0.070	0.158	0.116 (0.059)	0.074	0.130	0.152 (0.056)	0.071	0.162
	MI	−0.001 (0.047)	0.071	0.047	0.048 (0.045)	0.068	0.066	−0.002 (0.048)	0.072	0.048	0.051 (0.046)	0.068	0.069
	MIMP	−0.001 (0.047)	0.071	0.047	0.048 (0.045)	0.068	0.066	−0.002 (0.048)	0.072	0.048	0.052 (0.046)	0.069	0.069
	GBM	0.066 (0.050)	0.067	0.083	–	–	–	–	–	–	–	–	–
	GBM + SI + pe	0.039 (0.047)	0.067	0.061	–	–	–	–	–	–	–	–	–
**MAR2**	SI + pe + pu	− 0.009 (0.051)	0.070	0.052	0.040 (0.050)	0.068	0.064	−0.011 (0.053)	0.072	0.054	0.043 (0.050)	0.068	0.066
	SI + pe	−0.009 (0.051)	0.070	0.052	0.041 (0.049)	0.068	0.064	−0.010 (0.053)	0.072	0.054	0.045 (0.050)	0.068	0.067
	TMI	0.090 (0.069)	0.078	0.113	0.125 (0.066)	0.074	0.141	0.093 (0.071)	0.080	0.117	0.128 (0.067)	0.076	0.144
	MI	−0.008 (0.046)	0.071	0.047	0.041 (0.046)	0.068	0.062	−0.010 (0.048)	0.073	0.049	0.044 (0.046)	0.069	0.064
	MIMP	−0.008 (0.047)	0.072	0.048	0.042 (0.046)	0.068	0.062	−0.010 (0.049)	0.073	0.050	0.045 (0.047)	0.069	0.065
	GBM	0.058 (0.051)	0.067	0.077	–	–	–	–	–	–	–	–	–
	GBM + SI + pe	0.036 (0.048)	0.067	0.060	–	–	–	–	–	–	–	–	–
**MAR Sinister**	SI + pe + pu	−0.006 (0.050)	0.070	0.050	0.040 (0.048)	0.067	0.062	−0.007 (0.052)	0.071	0.052	0.042 (0.048)	0.068	0.064
	SI + pe	−0.012 (0.051)	0.071	0.052	0.039 (0.049)	0.068	0.063	−0.014 (0.053)	0.072	0.055	0.042 (0.050)	0.069	0.065
	TMI	0.113 (0.060)	0.075	0.128	0.147 (0.057)	0.072	0.158	0.113 (0.062)	0.077	0.129	0.151 (0.059)	0.073	0.162
	MI	− 0.007 (0.047)	0.071	0.048	0.043 (0.045)	0.068	0.062	−0.009 (0.048)	0.072	0.049	0.046 (0.046)	0.069	0.065
	MIMP	−0.007 (0.047)	0.071	0.048	0.043 (0.046)	0.068	0.063	−0.009 (0.049)	0.073	0.050	0.046 (0.046)	0.069	0.065
	GBM	0.068 (0.050)	0.067	0.084	–	–	–	–	–	–	–	–	–
	GBM + SI + pe	0.035 (0.048)	0.067	0.059	–	–	–	–	–	–	–	–	–

*Note.* Complete: logistic regression with complete data before introducing missingness; *comGBM* GBM with complete data before introducing missingness; *SI + pe + pu* single imputation + prediction error + parameter uncertainty; *SI + pe* single imputation + prediction error; *TMI* treatment mean imputation; *MI* multiple imputation (*m* = 20); *MIMP* multiple imputation missingness pattern (*m* = 20); *GBM* GBM with incomplete data; *GBM + SI + pe* GBM after single imputation + prediction error; *SD* standard deviation; *SE* standard error; *RMSE* root mean squared error

When only true confounders were included in the propensity score model, causal effect estimates based on propensity scores estimated from GBM were biased, with the largest bias observed when applying GBM directly to the incomplete data, and smaller bias when applying GBM to SI + PE data. Treatment mean imputation had the largest bias. SI + PE, SI + PE + PU, MI, and MIMP have little to no bias and actually have less bias than before the missingness was introduced. This may be a result of the bias due to excluding interactions counteracting that due to missingness.

As in Scenario A, SEs were largest for treatment mean imputation. GBM using the incomplete data and GBM with SI + PE had smaller SEs than the other approaches; these SEs were similar to those of GBM using the complete data before missingness was introduced. SI + PE, SI + PE + PU, MI, and MIMP had larger SEs than either GBM approach; these SEs were similar to those obtained when logistic regression was used to estimate propensity scores on the complete data prior to introducing missingness.

Variable inclusion strategies for the propensity score model affected the estimates in a similar way as in Scenario A. When true confounder *x*_1_ was omitted from the propensity score model, an increase in bias was observed for all approaches. When non-confounder *x*_5_ was included, there was no increase in bias but the SEs increased slightly. When true confounder *x*_1_ was omitted and non-confounder *x*_5_ was included, an even larger bias was observed. All types of propensity model misspecification led to increased RMSE estimates, especially when a true confounder was excluded from the propensity score model.

As in Scenario A, the bias for GBM using either the incomplete data or with SI + PE decreased substantially as the sample size increased (see supplemental materials). However, the bias for the other approaches did not decrease as the sample size increased. As expected, the SE and RMSE estimates decreased as the sample size increased.

When missingness increased from 25 to 50%, a larger bias was observed for GBM with incomplete data (see supplemental materials). The bias of GBM with SI + PE also increased somewhat. Very little bias was observed for SI + PE, SI + PE + PU, MI, and MIMP when the data were missing under MCAR, MAR2, or MAR sinister, but the bias was larger when the missingness mechanism was MAR1, with a larger magnitude than with 25% missingness. SI + PE + PU had a larger bias than other approaches when the missingness was MAR sinister with 50% missingness.

The bias of approaches using logistic regression in scenario G was from two possible resources: missing data and propensity score model misspecification due to excluding interaction and higher order terms. Therefore, additional simulations were conducted for scenario G using the correct propensity score model. Table 7 shows the results for scenario G with a correct logistic model and 25% missingness under the three different sample sizes. Except for treatment mean imputation, very little bias was observed when the data were MCAR, MAR2, or MAR sinister. When the missingness mechanism was MAR1, all approaches were slightly biased, with treatment mean imputation having the largest bias. The results for 50% missingness using the correct logistic regression model, including interactions and higher order terms, were similar (see supplementary materials).

**Table 7:** Simulation results for scenario G with the correct logistic model (25%missing)

Missingness Mechanism	Method	*n* = 500			*n* = 1000			*n* = 5000		
		Bias (SD)	SE	RMSE	Bias (SD)	SE	RMSE	Bias (SD)	SE	RMSE
	complete	−0.002 (0.045)	0.070	0.045	0.000 (0.030)	0.049	0.030	0.001 (0.014)	0.022	0.014
**MCAR**	SI + pe + pu	−0.002 (0.053)	0.070	0.053	−0.001 (0.035)	0.049	0.035	0.000 (0.016)	0.022	0.016
	SI + pe	−0.003 (0.052)	0.070	0.052	0.000 (0.035)	0.049	0.035	0.000 (0.016)	0.022	0.016
	TMI	−0.013 (0.062)	0.076	0.063	−0.009 (0.041)	0.053	0.042	−0.008 (0.018)	0.023	0.020
	MI	−0.002 (0.048)	0.071	0.048	−0.001 (0.033)	0.049	0.033	0.000 (0.015)	0.022	0.014
	MIMP	−0.002 (0.048)	0.071	0.049	−0.001 (0.033)	0.050	0.033	0.000 (0.015)	0.022	0.014
**MAR1**	SI + pe + pu	0.009 (0.052)	0.070	0.053	0.009 (0.035)	0.049	0.036	0.010 (0.016)	0.022	0.019
	SI + pe	0.008 (0.051)	0.070	0.052	0.010 (0.034)	0.049	0.035	0.011 (0.016)	0.022	0.019
	TMI	0.019 (0.057)	0.073	0.060	0.021 (0.039)	0.051	0.044	0.023 (0.018)	0.023	0.029
	MI	0.008 (0.047)	0.070	0.048	0.009 (0.031)	0.049	0.033	0.010 (0.015)	0.022	0.017
	MIMP	0.008 (0.048)	0.071	0.048	0.009 (0.032)	0.049	0.034	0.009 (0.015)	0.022	0.017
**MAR2**	SI + pe + pu	−0.001 (0.052)	0.070	0.052	0.003 (0.036)	0.049	0.036	0.002 (0.016)	0.022	0.016
	SI + pe	0.000 (0.052)	0.070	0.052	0.003 (0.035)	0.049	0.035	0.002 (0.016)	0.022	0.016
	TMI	−0.019 (0.064)	0.077	0.067	−0.015 (0.043)	0.054	0.046	−0.015 (0.019)	0.024	0.024
	MI	0.000 (0.047)	0.071	0.048	0.003 (0.032)	0.049	0.033	0.002 (0.015)	0.022	0.014
	MIMP	0.001 (0.047)	0.071	0.046	0.004 (0.033)	0.050	0.033	0.005 (0.015)	0.022	0.015
**MAR sinister**	SI + pe + pu	0.003 (0.050)	0.070	0.050	0.002 (0.035)	0.049	0.035	0.000 (0.016)	0.022	0.016
	SI + pe	−0.003 (0.052)	0.070	0.052	0.002 (0.035)	0.049	0.035	0.000 (0.016)	0.022	0.016
	TMI	0.002 (0.061)	0.076	0.061	0.003 (0.042)	0.052	0.042	−0.004 (0.018)	0.023	0.018
	MI	0.001 (0.047)	0.071	0.048	0.002 (0.033)	0.049	0.032	0.000 (0.015)	0.022	0.015
	MIMP	0.001 (0.047)	0.071	0.047	0.002 (0.033)	0.049	0.033	0.000 (0.015)	0.022	0.014

*Note.* Complete: logistic regression with complete data before introducing missingness; *SI + pe + pu* single imputation + prediction error + parameter uncertainty; *SI + pe* single imputation + prediction error; *TMI* treatment mean imputation; *MI* multiple imputation (m = 20); *MIMP* multiple imputation missingness pattern (m = 20); *SD* standard deviation; *SE* standard error; *RMSE* root mean squared error

## Discussion

The present study compared seven potentially useful approaches for missingness on the covariates used for estimating propensity scores. Results suggested that SI + PE, SI + PE + PU, MI, and MIMP have consistently good performance under Scenario A for all missingness mechanisms, sample sizes, and missing percentages. In contrast, treatment mean imputation was associated with a larger bias than the other approaches for all scenarios. This is inconsistent with other findings, [7] which suggested using treatment mean imputation when MI is not feasible. This discordance may be attributable to differences in the simulation study design: 1) the previous study had 12% missingness compared with 25% or 50% missingness in the present study; 2) the previous study imposed missing values on three covariates such that all were missing or none were. Treatment mean imputation could be viewed as a single imputation procedure in which the only covariate is the exposure group and there is no prediction error or parameter uncertainty added to the imputed values. Thus, the imputation model would not include other variables related to the missingness, such as *x*_5_, *x*_6_, *x*_8_, *x*_9_, *T,* and *Y* for the *MAR-sinister* condition. It is likely for this reason that it does not perform as well as the other approaches. In any case, we strongly recommend against using treatment mean imputation.

As expected, none of the approaches using logistic regression for propensity score estimation performed well in Scenario G. When the propensity score model included the true confounders, including interaction and higher-order terms, SI + PE, SI + PE + PU, MI, and MIMP produced unbiased estimates.

Including a non-confounder in the propensity score model did not increase bias but the estimate was not as efficient. In practice, it may be difficult to identify true confounders from a set of baseline covariates. Including non-confounder covariates has the benefit of reducing bias with the chance of slightly reducing efficiency. Our results are similar to previous simulation studies on variable selection in propensity score models with complete data [27].

The missingness mechanism played more of a role at smaller samples sizes, a larger missingness rate, and in Scenario G. The missingness mechanisms satisfied the assumptions of MI and MIMP, which assume MAR, but they differed in terms of the complexity of the MAR dependencies. However, the missingness mechanism did not play a large role, particularly in comparison with role of GBM vs. logistic regression.

We expected GBM, either with the incomplete data or SI + PE, to have similar performance to logistic regression in Scenario A and better performance than logistic regression in Scenario G. However, results suggested that applying GBM to the incomplete data for propensity score estimation resulted in a larger bias than logistic regression with any imputation approach except for treatment mean imputation in both Scenarios A and G. We suspect that this may be due to the lack of inclusion of *Y* in the surrogate split approach used by GBM. GBM with SI + PE does include information on *Y* and the bias did decrease, though not entirely.

Even when there was no missing data involved, the estimate using GBM was more biased than the estimate with logistic regression. This finding contradicts a previous study comparing the performance of logistic regression and GBM using a similar study design [3] when the covariates do not contain missing values. That study’s results showed that GBM was superior to logistic regression in both Scenarios A and G. This discrepancy could be explained by the weight trimming used in the present study. Lee et al. [3] did not trim weights although extreme weights were observed, with a higher percentage of extreme weights for logistic regression than GBM. Lee et al. [28] examined weight trimming with complete data using the same simulation design. They found that weight trimming decreased the bias in the case of logistic regression but that the bias for GBM did not decrease in any scenario when weights were trimmed. Future research should evaluate the effect of weight trimming on bias when there is missing data.

### Limitations

The conclusions drawn from the current study may not generalize to a scenario where the exposure, outcome, and covariates are correlated differently. For example, if the outcome is strongly correlated with the missingness, then it is possible that the GBM surrogate split approach may be even more biased for the MAR-2 and MAR-sinister conditions because in these conditions the missingness mechanism depended on the outcome but the outcome is not included in the surrogate split approach. In addition, we did not examine balance because doing so would have required resolving the issue of the most appropriate strategy when balance is obtained in some imputations but not others. We leave this assessment to future research.

The present simulation study did not include a condition for missingness on the outcome variable. If there is missingness on the outcome variable, then based on the missing data literature more generally, multiple imputation would be necessary to account for the uncertainty in the imputation of the outcome variable. We chose not to include a condition in the simulation study for missingness on the outcome because the real data we have worked with has often included planned missingness on some of the potential confounders or other intermittent missingness on the questionnaire items but the outcome data are present. Future simulations should examine whether MI or MIMP performs better when there is also missingness on the outcome.

A further direction for future research is a more comprehensive assessment of methods for including interaction terms in the imputation model. Recent research [29] has shown if the model is congenial, then multiple imputation via chained equations, and specifically the mice R package, using the just-another-variable approach is unbiased. The problem with some of the original simulations on the topic (e.g., [30]) is that the parametric models were not congenial with the analytic models. In other words, they used the defaults in the mice R package, which do not include interaction terms in the parametric models. These issues have not yet been sorted out either specifically to propensity score models or more generally, as van Buuren [18] describes a simulation study in which a newer approach, a rejection sampling method that creates congenial imputations, [31] appears to perform the best.

## Conclusions

In summary, the results suggest that missing values in the covariates should be imputed before fitting the propensity score model to obtain unbiased causal effect estimates. We had expected the standard errors for MI and MIMP to be uniformly larger because these approaches take into account both the uncertainty due to imputation and that due to propensity score estimation. However, the single imputation approaches, which take into account only uncertainty due to propensity score estimation, had similar standard errors. This finding could be due to our choice of 20 imputations given that previous research has shown that as the number of imputations increases, the standard errors decrease [32]. Given that our results did not indicate differences in bias or SEs between the multiple and single imputation approaches, the single imputation approaches may be preferred due to their simplicity. Among the multiple imputation approaches, we recommend the use of MI over MIMP due to the implementation issues of needing to combine missingness patterns with too few observations. We do not recommend using GBM with incomplete data using the surrogate split method. Rather, imputation should be done prior to using GBM. We also do not recommend using treatment mean imputation.

## Supplementary information

**Additional file 1: Table S1.** Simulation results for scenario A, *n* = 500, 50%missing. **Table S2.** Simulation results for scenario G, *n* = 500, 50%missing. **Table S3.** Simulation results for scenario A, *n* = 1000, 25%missing. **Table S4.** Simulation results for scenario G, *n* = 1000, 25%missing. **Table S5.** Simulation results for scenario A, *n* = 1000, 50%missing. **Table S6.** Simulation results for scenario G, *n* = 1000, 50%missing. **Table S7.** Simulation results for scenario A, *n* = 5000, 25%missing. **Table S8.** Simulation results for scenario G, *n* = 5000, 25%missing. **Table S9.** Simulation results for scenario A, *n* = 5000, 50%missing. **Table S10.** Simulation results for scenario G, *n* = 5000, 50%missing. **Table S11.** Simulation results for scenario G with the correct logistic model (50%missing).

## Data Availability

The code used to generate the data and conduct the simulation is available from the corresponding author upon request.

## Abbreviations

AASM: Average absolute standardized mean
GBM: Generalized Boosted Modeling
IPW: Inverse probability weighting
MCAR: Missing completely at random
MAR: Missing at random
MNAR: Missing not at random
MI: Multiple imputation
MP: Missingness pattern
MIMP: Multiple imputation with missingness pattern
RMSE: Root mean squared error
SD: Standard deviation
SE: Standard error
SI: Single imputation
SI + PE: SI + prediction error
SI + PE + PU: SI + PE + parameter uncertainty

## References

[1] Rosenbaum PR, Rubin DB (1983). The central role of the propensity score in observational studies for causal effects. Biometrika.

[2] McCaffrey DF, Ridgeway G, Morral AR (2004). Propensity score estimation with boosted regression for evaluating causal effects in observational studies. Psychol Methods.

[3] Lee BK, Lessler J, Stuart EA (2010). Improving propensity score weighting using machine learning. Stat Med.

[4] Leyrat C, Seaman SR, White IR, Douglas I, Smeeth L, Kim J, Resche-Rigon M, Carpenter JR, Williamson EJ (2019). Propensity score analysis with partially observed covariates: how should multiple imputation be used?. Stat Methods Med Res.

[5] Mitra R, Reiter JP (2011). Estimating propensity scores with missing covariate data using general location mixture models. Stat Med.

[6] Mitra R, Reiter JP (2016). A comparison of two methods of estimating propensity scores after multiple imputation. Stat Methods Med Res.

[7] Crowe BJ, Lipkovich IA, Wang O (2010). Comparison of several imputation methods for missing baseline data in propensity scores analysis of binary outcome. Pharm Stat.

[8] Qu Y, Lipkovich I (2010). Propensity scoring with missing values. Analysis of Observational Health Care Data Using SAS.

[9] D’Agostino R, Lang W, Walkup M, Morgon T (2001). Examining the impact of missing data on propensity score estimation in determining the effectiveness of self-monitoring of blood glucose (SMBG). Health Serv Outcome Res Methodol.

[10] Rosenbaum PR, Rubin DB (1984). Reducing bias in observational studies using subclassification on the propensity score. J Am Stat Assoc.

[11] D'Agostino RB, Rubin DB (2000). Estimating and using propensity scores with partially missing data. J Am Stat Assoc.

[12] Qu Y, Lipkovich I (2009). Propensity score estimation with missing values using a multiple imputation missingness pattern (MIMP) approach. Stat Med.

[13] Rubin DB (1987). Multiple imputation for nonresponse in surveys.

[14] Schafer JL (1997). Analysis of incomplete multivariate data.

[15] Little RJA, Rubin DB (2002). Statistical analysis with missing data.

[16] van Buuren S (2007). Multiple imputation of discrete and continuous data by fully conditional specification. Stat Methods Med Res.

[17] van Buuren S, Groothuis-Oudshoorn K (2011). mice: Multivariate Imputation by Chained Equations in R. J Stat Software.

[18] Van Buuren S (2012). Flexible imputation of missing data.

[19] Cham H, West SG (2016). Propensity score analysis with missing data. Psychol Methods.

[20] Hayes JR, Groner JI (2008). Using multiple imputation and propensity scores to test the effect of car seats and seat belt usage on injury severity from trauma registry data. J Pediatr Surg.

[21] Robins JM, Hernan MA, Brumback B (2000). Marginal structural models and causal inference in epidemiology. Epidemiology.

[22] Setoguchi S, Schneeweiss S, Brookhart MA, Glynn RJ, Cook EF (2008). Evaluating uses of data mining techniques in propensity score estimation: a simulation study. Pharmacoepidemiol Drug Saf.

[23] Graham JW, Taylor BJ, Olchowski AE, Cumsille PE (2006). Planned missing data designs in psychological research. Psychol Methods.

[24] Collins LM, Schafer JL, Kam CM (2001). A comparison of inclusive and restrictive missing-data strategies in modern missing-data procedures. Psychol Methods.

[25] Allison PD (2002). Missing data: quantitative applications in the social sciences. Br J Math Stat Psychol.

[26] Ridgeway G, McCaffrey DF, Morral AR, Burgette L, Griffin BA (2012). Toolkit for weighting and analysis of nonequivalent groups: a tutorial for the twang package.

[27] Brookhart MA, Schneeweiss S, Rothman KJ, Glynn RJ, Avorn J, Stürmer T (2006). Variable selection for propensity score models. Am J Epidemiol.

[28] Lee BK, Lessler J, Stuart EA (2011). Weight trimming and propensity score weighting. PLoS One.

[29] Slade E, Naylor MG (2020). A fair comparison of tree-based and parametric methods in multiple imputation by chained equations. Stat Med.

[30] Doove LL, Van Buuren S, Dusseldorp E (2014). Recursive partitioning for missing data imputation in the presence of interaction effects. Comput Stat Data Anal.

[31] Bartlett JW, Seaman SR, White IR, Carpenter JR (2015). Alzheimer's disease neuroimaging I: multiple imputation of covariates by fully conditional specification: accommodating the substantive model. Stat Methods Med Res.

[32] Graham JW, Olchowski AE, Gilreath TD (2007). How many imputations are really needed? Some practical clarifications of multiple imputation theory. Prev Sci.

