# Supplementary material for: Comparison of methods for handling covariate missingness in propensity score estimation with a binary exposure
Source: BMC Med Res Methodol. 2020 Jun 26;20:168. doi: 10.1186/s12874-020-01053-4 (PMC7318364; doi:10.1186/s12874-020-01053-4)
Supplement: Supplementary file 1 — Additional file 1: Table S1. Simulation results for scenario A, n = 500, 50%missing. Table S2. Simulation results for scenario G, n = 500, 50%missing. Table S3. Simulation results for scenario A, n = 1000, 25%missing. Table S4. Simulation results for scenario G, n = 1000, 25%missing. Table S5. Simulation results for scenario A, n = 1000, 50%missing. Table S6. Simulation results for scenario G, n = 1000, 50%missing. Table S7. Simulation results for scenario A, n = 5000, 25%missing. Table S8. Simulation results for scenario G, n = 5000, 25%missing. Table S9. Simulation results for scenario A, n = 5000, 50%missing. Table S10. Simulation results for scenario G, n = 5000, 50%missing. Table S11. Simulation results for scenario G with the correct logistic model (50%missing). [file 12874_2020_1053_MOESM1_ESM.docx]

**Supplementary Materials**

*Note.* **Complete**: logistic regression with complete data before introducing missingness; **comGBM**: GBM with complete data before introducing missingness; **SI + pe + pu:** single imputation + prediction error + parameter uncertainty; **SI + pe**: single imputation + prediction error; **TMI**: treatment mean imputation; **MI**: multiple imputation (m = 20); **MIMP**: multiple imputation missingness pattern (m = 20); **GBM**: GBM with incomplete data; **GBM + SI + pe**: GBM after single imputation + prediction error; **SD**: standard deviation; **SE**: standard error; **RMSE**: root mean squared error.

## Table 1. Simulation results for scenario A, n = 500, 50%missing.

| **Missingness Mechanism** | **Method** | **True confounders** | | | **Leave x1 out** | | | **Add x5** | | | **Leave x1 out + Add x5** | | |
| --- | --- | --- | --- | --- | --- | --- | --- | --- | --- | --- | --- | --- | --- |
|  |  | Bias (SD) | SE | RMSE | Bias (SD) | SE | RMSE | Bias (SD) | SE | RMSE | Bias (SD) | SE | RMSE |
|  | complete | -0.001 (0.041) | 0.068 | 0.041 | 0.036 (0.043) | 0.067 | 0.056 | -0.001 (0.043) | 0.070 | 0.043 | 0.042 (0.043) | 0.068 | 0.060 |
|  | comGBM | 0.029 (0.042) | 0.067 | 0.051 | - | - | - | - | - | - | - | - | - |
| **MCAR** | SI + pe + pu | 0.002 (0.067) | 0.068 | 0.067 | 0.037 (0.064) | 0.067 | 0.074 | 0.002 (0.069) | 0.070 | 0.069 | 0.043 (0.065) | 0.068 | 0.078 |
|  | SI + pe | 0.000 (0.061) | 0.069 | 0.061 | 0.037 (0.059) | 0.067 | 0.070 | 0.001 (0.063) | 0.070 | 0.063 | 0.043 (0.060) | 0.068 | 0.074 |
|  | TMI | 0.149 (0.142) | 0.090 | 0.206 | 0.164 (0.141) | 0.089 | 0.216 | 0.156 (0.144) | 0.092 | 0.212 | 0.173 (0.143) | 0.090 | 0.224 |
|  | MI | 0.002 (0.054) | 0.070 | 0.054 | 0.037 (0.053) | 0.068 | 0.065 | 0.002 (0.055) | 0.072 | 0.055 | 0.043 (0.054) | 0.069 | 0.069 |
|  | MIMP | 0.002 (0.055) | 0.070 | 0.055 | 0.037 (0.054) | 0.068 | 0.065 | 0.002 (0.056) | 0.072 | 0.056 | 0.043 (0.055) | 0.069 | 0.070 |
|  | GBM | 0.102 (0.056) | 0.066 | 0.116 | - | - | - | - | - | - | - | - | - |
|  | GBM + SI + pe | 0.042 (0.054) | 0.066 | 0.068 | - | - | - | - | - | - | - | - | - |
| **MAR1** | SI + pe + pu | 0.000 (0.061) | 0.068 | 0.061 | 0.035 (0.057) | 0.067 | 0.067 | 0.000 (0.062) | 0.070 | 0.062 | 0.042 (0.058) | 0.068 | 0.072 |
|  | SI + pe | -0.001 (0.058) | 0.069 | 0.058 | 0.035 (0.055) | 0.067 | 0.065 | -0.001 (0.059) | 0.070 | 0.059 | 0.041 (0.056) | 0.068 | 0.069 |
|  | TMI | 0.171 (0.115) | 0.079 | 0.206 | 0.189 (0.114) | 0.078 | 0.221 | 0.182 (0.116) | 0.080 | 0.216 | 0.198 (0.115) | 0.079 | 0.229 |
|  | MI | 0.001 (0.051) | 0.070 | 0.051 | 0.036 (0.050) | 0.068 | 0.062 | 0.001 (0.053) | 0.071 | 0.053 | 0.042 (0.051) | 0.069 | 0.066 |
|  | MIMP | 0.002 (0.051) | 0.070 | 0.051 | 0.038 (0.050) | 0.068 | 0.063 | 0.001 (0.053) | 0.072 | 0.053 | 0.043 (0.051) | 0.069 | 0.067 |
|  | GBM | 0.099 (0.053) | 0.066 | 0.112 | - | - | - | - | - | - | - | - | - |
|  | GBM + SI + pe | 0.040 (0.050) | 0.066 | 0.064 | - | - | - | - | - | - | - | - | - |
| **MAR2** | SI + pe + pu | 0.003 (0.064) | 0.069 | 0.064 | 0.037 (0.062) | 0.067 | 0.072 | 0.004 (0.066) | 0.070 | 0.066 | 0.043 (0.064) | 0.068 | 0.077 |
|  | SI + pe | 0.003 (0.061) | 0.068 | 0.061 | 0.037 (0.060) | 0.067 | 0.070 | 0.004 (0.063) | 0.070 | 0.063 | 0.043 (0.061) | 0.068 | 0.075 |
|  | TMI | 0.156 (0.146) | 0.097 | 0.214 | 0.168 (0.141) | 0.095 | 0.219 | 0.165 (0.151) | 0.099 | 0.224 | 0.176 (0.145) | 0.097 | 0.228 |
|  | MI | 0.004 (0.054) | 0.070 | 0.054 | 0.037 (0.054) | 0.068 | 0.065 | 0.005 (0.056) | 0.072 | 0.056 | 0.043 (0.055) | 0.069 | 0.070 |
|  | MIMP | 0.009 (0.056) | 0.071 | 0.057 | 0.043 (0.055) | 0.069 | 0.070 | 0.007 (0.057) | 0.072 | 0.057 | 0.046 (0.056) | 0.070 | 0.072 |
|  | GBM | 0.115 (0.057) | 0.067 | 0.128 | - | - | - | - | - | - | - | - | - |
|  | GBM + SI + pe | 0.048 (0.054) | 0.067 | 0.072 | - | - | - | - | - | - | - | - | - |
| **MAR Sinister** | SI + pe + pu | 0.021 (0.058) | 0.067 | 0.062 | 0.044 (0.058) | 0.067 | 0.073 | 0.022 (0.059) | 0.069 | 0.063 | 0.048 (0.059) | 0.068 | 0.076 |
|  | SI + pe | 0.005 (0.060) | 0.068 | 0.060 | 0.040 (0.058) | 0.067 | 0.070 | 0.005 (0.062) | 0.070 | 0.062 | 0.046 (0.059) | 0.068 | 0.075 |
|  | TMI | 0.185 (0.126) | 0.085 | 0.224 | 0.200 (0.124) | 0.084 | 0.235 | 0.192 (0.129) | 0.087 | 0.231 | 0.208 (0.127) | 0.086 | 0.244 |
|  | MI | 0.013 (0.052) | 0.069 | 0.054 | 0.045 (0.051) | 0.068 | 0.068 | 0.014 (0.053) | 0.071 | 0.055 | 0.052 (0.052) | 0.069 | 0.074 |
|  | MIMP | 0.013 (0.052) | 0.070 | 0.054 | 0.045 (0.051) | 0.068 | 0.068 | 0.015 (0.053) | 0.071 | 0.055 | 0.052 (0.052) | 0.069 | 0.074 |
|  | GBM | 0.111 (0.055) | 0.066 | 0.124 | - | - | - | - | - | - | - | - | - |
|  | GBM + SI + pe | 0.046 (0.053) | 0.066 | 0.070 | - | - | - | - | - | - | - | - | - |

**Table 2. Simulation results for scenario G, n = 500, 50%missing.**

| **Missingness Mechanism** | **Method** | **True confounders** | | | **Leave x1 out** | | | **Add x5** | | | **Leave x1 out + Add x5** | | |
| --- | --- | --- | --- | --- | --- | --- | --- | --- | --- | --- | --- | --- | --- |
|  |  | Bias (SD) | SE | RMSE | Bias (SD) | SE | RMSE | Bias (SD) | SE | RMSE | Bias (SD) | SE | RMSE |
|  | complete | -0.014 (0.044) | 0.071 | 0.046 | 0.037 (0.043) | 0.068 | 0.057 | -0.016 (0.045) | 0.073 | 0.048 | 0.040 (0.043) | 0.069 | 0.059 |
|  | comGBM | 0.029 (0.044) | 0.067 | 0.053 | - | - | - | - | - | - | - | - | - |
| **MCAR** | SI + pe + pu | -0.006 (0.068) | 0.070 | 0.068 | 0.042 (0.064) | 0.067 | 0.077 | -0.007 (0.070) | 0.072 | 0.070 | 0.046 (0.064) | 0.068 | 0.079 |
|  | SI + pe | -0.007 (0.062) | 0.070 | 0.062 | 0.042 (0.059) | 0.068 | 0.072 | -0.008 (0.065) | 0.072 | 0.065 | 0.046 (0.060) | 0.068 | 0.076 |
|  | TMI | 0.159 (0.129) | 0.093 | 0.205 | 0.180 (0.124) | 0.091 | 0.219 | 0.161 (0.133) | 0.095 | 0.209 | 0.183 (0.128) | 0.093 | 0.223 |
|  | MI | -0.006 (0.056) | 0.072 | 0.056 | 0.041 (0.053) | 0.069 | 0.067 | -0.007 (0.057) | 0.074 | 0.057 | 0.045 (0.054) | 0.069 | 0.070 |
|  | MIMP | -0.006 (0.056) | 0.072 | 0.056 | 0.042 (0.054) | 0.069 | 0.068 | -0.007 (0.057) | 0.074 | 0.057 | 0.045 (0.054) | 0.070 | 0.070 |
|  | GBM | 0.086 (0.058) | 0.067 | 0.104 | - | - | - | - | - | - | - | - | - |
|  | GBM + SI + pe | 0.036 (0.054) | 0.067 | 0.065 | - | - | - | - | - | - | - | - | - |
| **MAR1** | SI + pe + pu | 0.021 (0.063) | 0.069 | 0.066 | 0.067 (0.057) | 0.067 | 0.088 | 0.020 (0.064) | 0.071 | 0.067 | 0.071 (0.057) | 0.067 | 0.091 |
|  | SI + pe | 0.022 (0.059) | 0.069 | 0.063 | 0.067 (0.055) | 0.067 | 0.087 | 0.021 (0.060) | 0.071 | 0.064 | 0.071 (0.055) | 0.067 | 0.090 |
|  | TMI | 0.242 (0.095) | 0.077 | 0.260 | 0.264 (0.093) | 0.076 | 0.280 | 0.248 (0.096) | 0.078 | 0.266 | 0.268 (0.093) | 0.077 | 0.284 |
|  | MI | 0.022 (0.053) | 0.070 | 0.057 | 0.068 (0.050) | 0.068 | 0.084 | 0.021 (0.054) | 0.072 | 0.058 | 0.072 (0.050) | 0.068 | 0.088 |
|  | MIMP | 0.022 (0.053) | 0.071 | 0.057 | 0.068 (0.050) | 0.068 | 0.084 | 0.021 (0.054) | 0.072 | 0.058 | 0.072 (0.051) | 0.069 | 0.088 |
|  | GBM | 0.098 (0.052) | 0.066 | 0.111 | - | - | - | - | - | - | - | - | - |
|  | GBM + SI + pe | 0.054 (0.050) | 0.066 | 0.074 | - | - | - | - | - | - | - | - | - |
| **MAR2** | SI + pe + pu | 0.002 (0.067) | 0.070 | 0.067 | 0.045 (0.063) | 0.068 | 0.077 | 0.000 (0.068) | 0.072 | 0.068 | 0.049 (0.063) | 0.068 | 0.080 |
|  | SI + pe | -0.001 (0.064) | 0.070 | 0.064 | 0.044 (0.061) | 0.068 | 0.075 | -0.003 (0.066) | 0.072 | 0.066 | 0.047 (0.062) | 0.068 | 0.078 |
|  | TMI | 0.142 (0.156) | 0.103 | 0.211 | 0.158 (0.146) | 0.100 | 0.215 | 0.146 (0.157) | 0.104 | 0.214 | 0.162 (0.147) | 0.101 | 0.219 |
|  | MI | 0.002 (0.056) | 0.072 | 0.056 | 0.046 (0.055) | 0.069 | 0.072 | 0.001 (0.058) | 0.074 | 0.058 | 0.050 (0.056) | 0.070 | 0.075 |
|  | MIMP | 0.005 (0.057) | 0.073 | 0.057 | 0.050 (0.056) | 0.069 | 0.075 | 0.002 (0.059) | 0.074 | 0.059 | 0.051 (0.056) | 0.070 | 0.076 |
|  | GBM | 0.095 (0.058) | 0.067 | 0.111 | - | - | - | - | - | - | - | - | - |
|  | GBM + SI + pe | 0.042 (0.055) | 0.067 | 0.069 | - | - | - | - | - | - | - | - | - |
| **MAR Sinister** | SI + pe + pu | 0.014 (0.061) | 0.069 | 0.063 | 0.051 (0.060) | 0.067 | 0.079 | 0.013 (0.063) | 0.070 | 0.064 | 0.053 (0.061) | 0.068 | 0.081 |
|  | SI + pe | 0.000 (0.065) | 0.070 | 0.065 | 0.049 (0.060) | 0.068 | 0.077 | -0.001 (0.066) | 0.072 | 0.066 | 0.053 (0.061) | 0.068 | 0.081 |
|  | TMI | 0.199 (0.117) | 0.089 | 0.231 | 0.220 (0.114) | 0.087 | 0.248 | 0.201 (0.122) | 0.091 | 0.235 | 0.224 (0.118) | 0.088 | 0.253 |
|  | MI | 0.007 (0.056) | 0.072 | 0.056 | 0.051 (0.054) | 0.069 | 0.074 | 0.006 (0.057) | 0.073 | 0.057 | 0.055 (0.054) | 0.069 | 0.077 |
|  | MIMP | 0.006 (0.056) | 0.072 | 0.056 | 0.051 (0.054) | 0.069 | 0.074 | 0.006 (0.057) | 0.073 | 0.057 | 0.055 (0.054) | 0.070 | 0.077 |
|  | GBM | 0.096 (0.057) | 0.066 | 0.112 | - | - | - | - | - | - | - | - | - |
|  | GBM + SI + pe | 0.041 (0.055) | 0.067 | 0.069 | - | - | - | - | - | - | - | - | - |

## Table 3. Simulation results for scenario A, n = 1000, 25%missing.

| **Missingness Mechanism** | **Method** | **True confounders** | | | **Leave x1 out** | | | **Add x5** | | | **Leave x1 out + Add x5** | | |
| --- | --- | --- | --- | --- | --- | --- | --- | --- | --- | --- | --- | --- | --- |
|  |  | Bias (SD) | SE | RMSE | Bias (SD) | SE | RMSE | Bias (SD) | SE | RMSE | Bias (SD) | SE | RMSE |
|  | complete | 0.000 (0.028) | 0.048 | 0.028 | 0.037 (0.028) | 0.047 | 0.046 | 0.000 (0.029) | 0.049 | 0.029 | 0.044 (0.029) | 0.048 | 0.053 |
|  | comGBM | 0.025 (0.028) | 0.047 | 0.038 | - | - | - | - | - | - | - | - | - |
| **MCAR** | SI + pe + pu | 0.000 (0.034) | 0.048 | 0.034 | 0.037 (0.033) | 0.047 | 0.050 | 0.000 (0.035) | 0.049 | 0.035 | 0.044 (0.034) | 0.048 | 0.056 |
|  | SI + pe | 0.000 (0.033) | 0.048 | 0.033 | 0.038 (0.033) | 0.047 | 0.050 | 0.000 (0.034) | 0.049 | 0.034 | 0.044 (0.034) | 0.048 | 0.056 |
|  | TMI | 0.113 (0.040) | 0.051 | 0.120 | 0.139 (0.040) | 0.050 | 0.145 | 0.117 (0.041) | 0.052 | 0.124 | 0.146 (0.041) | 0.051 | 0.152 |
|  | MI | 0.000 (0.031) | 0.048 | 0.031 | 0.037 (0.031) | 0.047 | 0.048 | 0.000 (0.032) | 0.050 | 0.032 | 0.044 (0.032) | 0.048 | 0.054 |
|  | MIMP | -0.001 (0.031) | 0.049 | 0.031 | 0.037 (0.031) | 0.047 | 0.048 | -0.001 (0.032) | 0.050 | 0.032 | 0.043 (0.032) | 0.048 | 0.054 |
|  | GBM | 0.063 (0.033) | 0.047 | 0.071 | - | - | - | - | - | - | - | - | - |
|  | GBM + SI + pe | 0.031 (0.031) | 0.047 | 0.044 | - | - | - | - | - | - | - | - | - |
| **MAR1** | SI + pe + pu | 0.000 (0.034) | 0.048 | 0.034 | 0.037 (0.033) | 0.047 | 0.050 | 0.000 (0.035) | 0.049 | 0.035 | 0.044 (0.034) | 0.048 | 0.056 |
|  | SI + pe | 0.000 (0.033) | 0.048 | 0.033 | 0.037 (0.033) | 0.047 | 0.050 | 0.001 (0.034) | 0.049 | 0.034 | 0.044 (0.033) | 0.048 | 0.055 |
|  | TMI | 0.110 (0.036) | 0.049 | 0.116 | 0.137 (0.036) | 0.048 | 0.142 | 0.116 (0.038) | 0.050 | 0.122 | 0.144 (0.037) | 0.049 | 0.149 |
|  | MI | 0.000 (0.030) | 0.048 | 0.030 | 0.037 (0.030) | 0.047 | 0.048 | 0.000 (0.031) | 0.049 | 0.031 | 0.044 (0.031) | 0.048 | 0.054 |
|  | MIMP | 0.000 (0.031) | 0.048 | 0.031 | 0.038 (0.031) | 0.047 | 0.049 | 0.000 (0.032) | 0.050 | 0.032 | 0.044 (0.031) | 0.048 | 0.054 |
|  | GBM | 0.063 (0.032) | 0.047 | 0.071 | - | - | - | - | - | - | - | - | - |
|  | GBM + SI + pe | 0.031 (0.031) | 0.047 | 0.044 | - | - | - | - | - | - | - | - | - |
| **MAR2** | SI + pe + pu | 0.003 (0.034) | 0.048 | 0.034 | 0.040 (0.033) | 0.047 | 0.052 | 0.003 (0.035) | 0.049 | 0.035 | 0.046 (0.034) | 0.048 | 0.057 |
|  | SI + pe | 0.002 (0.034) | 0.048 | 0.034 | 0.039 (0.033) | 0.047 | 0.051 | 0.002 (0.035) | 0.049 | 0.035 | 0.045 (0.034) | 0.048 | 0.056 |
|  | TMI | 0.114 (0.040) | 0.052 | 0.121 | 0.140 (0.039) | 0.050 | 0.145 | 0.121 (0.042) | 0.053 | 0.128 | 0.148 (0.040) | 0.051 | 0.153 |
|  | MI | 0.002 (0.031) | 0.048 | 0.031 | 0.039 (0.031) | 0.047 | 0.050 | 0.003 (0.032) | 0.049 | 0.032 | 0.046 (0.031) | 0.048 | 0.055 |
|  | MIMP | 0.004 (0.031) | 0.049 | 0.031 | 0.042 (0.031) | 0.048 | 0.052 | 0.004 (0.032) | 0.050 | 0.032 | 0.047 (0.031) | 0.048 | 0.056 |
|  | GBM | 0.066 (0.034) | 0.047 | 0.074 | - | - | - | - | - | - | - | - | - |
|  | GBM + SI + pe | 0.034 (0.032) | 0.047 | 0.047 | - | - | - | - | - | - | - | - | - |
| **MAR Sinister** | SI + pe + pu | 0.003 (0.034) | 0.048 | 0.034 | 0.036 (0.033) | 0.047 | 0.049 | 0.003 (0.035) | 0.049 | 0.035 | 0.042 (0.033) | 0.048 | 0.053 |
|  | SI + pe | 0.002 (0.034) | 0.048 | 0.034 | 0.040 (0.033) | 0.047 | 0.052 | 0.003 (0.035) | 0.049 | 0.035 | 0.047 (0.034) | 0.048 | 0.058 |
|  | TMI | 0.125 (0.040) | 0.050 | 0.131 | 0.151 (0.039) | 0.049 | 0.156 | 0.129 (0.041) | 0.051 | 0.135 | 0.159 (0.040) | 0.050 | 0.164 |
|  | MI | 0.002 (0.032) | 0.048 | 0.032 | 0.039 (0.031) | 0.047 | 0.050 | 0.003 (0.033) | 0.049 | 0.033 | 0.045 (0.032) | 0.048 | 0.055 |
|  | MIMP | 0.002 (0.032) | 0.048 | 0.032 | 0.039 (0.032) | 0.047 | 0.050 | 0.002 (0.033) | 0.050 | 0.033 | 0.045 (0.032) | 0.048 | 0.055 |
|  | GBM | 0.069 (0.033) | 0.047 | 0.076 | - | - | - | - | - | - | - | - | - |
|  | GBM + SI + pe | 0.033 (0.032) | 0.047 | 0.046 | - | - | - | - | - | - | - | - | - |

## Table 4. Simulation results for scenario G, n = 1000, 25%missing.

| **Missingness Mechanism** | **Method** | **True confounders** | | | **Leave x1 out** | | | **Add x5** | | | **Leave x1 out + Add x5** | | |
| --- | --- | --- | --- | --- | --- | --- | --- | --- | --- | --- | --- | --- | --- |
|  |  | Bias (SD) | SE | RMSE | Bias (SD) | SE | RMSE | Bias (SD) | SE | RMSE | Bias (SD) | SE | RMSE |
|  | complete | -0.012 (0.029) | 0.050 | 0.031 | 0.040 (0.029) | 0.048 | 0.049 | -0.014 (0.030) | 0.051 | 0.033 | 0.043 (0.029) | 0.048 | 0.052 |
|  | comGBM | 0.027 (0.030) | 0.048 | 0.040 | - | - | - | - | - | - | - | - | - |
| **MCAR** | SI + pe + pu | -0.010 (0.035) | 0.050 | 0.036 | 0.041 (0.034) | 0.048 | 0.053 | -0.011 (0.036) | 0.051 | 0.038 | 0.045 (0.034) | 0.048 | 0.056 |
|  | SI + pe | -0.009 (0.035) | 0.050 | 0.036 | 0.042 (0.034) | 0.048 | 0.054 | -0.011 (0.036) | 0.051 | 0.038 | 0.046 (0.034) | 0.048 | 0.057 |
|  | TMI | 0.100 (0.043) | 0.054 | 0.109 | 0.135 (0.041) | 0.052 | 0.141 | 0.101 (0.044) | 0.055 | 0.110 | 0.139 (0.041) | 0.052 | 0.145 |
|  | MI | -0.010 (0.033) | 0.050 | 0.034 | 0.041 (0.032) | 0.048 | 0.052 | -0.012 (0.033) | 0.051 | 0.035 | 0.045 (0.032) | 0.048 | 0.055 |
|  | MIMP | -0.011 (0.033) | 0.050 | 0.035 | 0.041 (0.032) | 0.048 | 0.052 | -0.012 (0.034) | 0.051 | 0.036 | 0.045 (0.032) | 0.049 | 0.055 |
|  | GBM | 0.057 (0.034) | 0.047 | 0.066 | - | - | - | - | - | - | - | - | - |
|  | GBM + SI + pe | 0.031 (0.033) | 0.047 | 0.045 | - | - | - | - | - | - | - | - | - |
| **MAR1** | SI + pe + pu | 0.000 (0.034) | 0.049 | 0.034 | 0.051 (0.033) | 0.047 | 0.061 | -0.001 (0.035) | 0.050 | 0.035 | 0.054 (0.033) | 0.048 | 0.063 |
|  | SI + pe | 0.001 (0.034) | 0.049 | 0.034 | 0.051 (0.033) | 0.047 | 0.061 | 0.000 (0.035) | 0.050 | 0.035 | 0.055 (0.033) | 0.048 | 0.064 |
|  | TMI | 0.117 (0.039) | 0.051 | 0.123 | 0.153 (0.037) | 0.049 | 0.157 | 0.120 (0.039) | 0.052 | 0.126 | 0.156 (0.038) | 0.050 | 0.161 |
|  | MI | 0.000 (0.031) | 0.050 | 0.031 | 0.051 (0.031) | 0.048 | 0.060 | -0.001 (0.032) | 0.051 | 0.032 | 0.054 (0.031) | 0.048 | 0.062 |
|  | MIMP | 0.000 (0.031) | 0.050 | 0.031 | 0.051 (0.031) | 0.048 | 0.060 | -0.001 (0.032) | 0.051 | 0.032 | 0.055 (0.031) | 0.048 | 0.063 |
|  | GBM | 0.064 (0.033) | 0.047 | 0.072 | - | - | - | - | - | - | - | - | - |
|  | GBM + SI + pe | 0.037 (0.032) | 0.047 | 0.049 | - | - | - | - | - | - | - | - | - |
| **MAR2** | SI + pe + pu | -0.005 (0.035) | 0.050 | 0.035 | 0.045 (0.034) | 0.048 | 0.056 | -0.007 (0.036) | 0.051 | 0.037 | 0.049 (0.034) | 0.048 | 0.060 |
|  | SI + pe | -0.005 (0.035) | 0.050 | 0.035 | 0.045 (0.033) | 0.048 | 0.056 | -0.007 (0.036) | 0.051 | 0.037 | 0.049 (0.034) | 0.048 | 0.060 |
|  | TMI | 0.098 (0.045) | 0.055 | 0.108 | 0.133 (0.043) | 0.052 | 0.140 | 0.101 (0.046) | 0.056 | 0.111 | 0.136 (0.043) | 0.053 | 0.143 |
|  | MI | -0.005 (0.032) | 0.050 | 0.032 | 0.045 (0.031) | 0.048 | 0.055 | -0.007 (0.033) | 0.051 | 0.034 | 0.048 (0.032) | 0.048 | 0.058 |
|  | MIMP | -0.004 (0.033) | 0.050 | 0.033 | 0.047 (0.032) | 0.048 | 0.057 | -0.006 (0.034) | 0.051 | 0.035 | 0.049 (0.032) | 0.049 | 0.059 |
|  | GBM | 0.058 (0.035) | 0.047 | 0.068 | - | - | - | - | - | - | - | - | - |
|  | GBM + SI + pe | 0.034 (0.033) | 0.047 | 0.047 | - | - | - | - | - | - | - | - | - |
| **MAR Sinister** | SI + pe + pu | -0.006 (0.035) | 0.049 | 0.036 | 0.039 (0.033) | 0.048 | 0.051 | -0.008 (0.035) | 0.050 | 0.036 | 0.042 (0.033) | 0.048 | 0.053 |
|  | SI + pe | -0.008 (0.035) | 0.050 | 0.036 | 0.044 (0.033) | 0.048 | 0.055 | -0.009 (0.036) | 0.051 | 0.037 | 0.047 (0.034) | 0.048 | 0.058 |
|  | TMI | 0.113 (0.043) | 0.053 | 0.121 | 0.148 (0.041) | 0.051 | 0.154 | 0.114 (0.044) | 0.054 | 0.122 | 0.152 (0.042) | 0.052 | 0.158 |
|  | MI | -0.007 (0.032) | 0.050 | 0.033 | 0.043 (0.031) | 0.048 | 0.053 | -0.009 (0.033) | 0.051 | 0.034 | 0.046 (0.031) | 0.048 | 0.055 |
|  | MIMP | -0.007 (0.033) | 0.050 | 0.034 | 0.043 (0.031) | 0.048 | 0.053 | -0.008 (0.033) | 0.051 | 0.034 | 0.047 (0.031) | 0.048 | 0.056 |
|  | GBM | 0.064 (0.035) | 0.047 | 0.073 | - | - | - | - | - | - | - | - | - |
|  | GBM + SI + pe | 0.031 (0.033) | 0.047 | 0.045 | - | - | - | - | - | - | - | - | - |

## Table 5. Simulation results for scenario A, n = 1000, 50%missing.

| **Missingness Mechanism** | **Method** | **True confounders** | | | **Leave x1 out** | | | **Add x5** | | | **Leave x1 out + Add x5** | | |
| --- | --- | --- | --- | --- | --- | --- | --- | --- | --- | --- | --- | --- | --- |
|  |  | Bias (SD) | SE | RMSE | Bias (SD) | SE | RMSE | Bias (SD) | SE | RMSE | Bias (SD) | SE | RMSE |
|  | complete | 0.000 (0.028) | 0.048 | 0.028 | 0.037 (0.028) | 0.047 | 0.046 | 0.000 (0.029) | 0.049 | 0.029 | 0.044 (0.029) | 0.048 | 0.053 |
|  | comGBM | 0.025 (0.028) | 0.047 | 0.038 | - | - | - | - | - | - | - | - | - |
| **MCAR** | SI + pe + pu | 0.003 (0.045) | 0.048 | 0.045 | 0.039 (0.043) | 0.047 | 0.058 | 0.003 (0.046) | 0.049 | 0.046 | 0.045 (0.044) | 0.048 | 0.063 |
|  | SI + pe | 0.000 (0.043) | 0.048 | 0.043 | 0.037 (0.042) | 0.047 | 0.056 | 0.001 (0.045) | 0.049 | 0.045 | 0.044 (0.043) | 0.048 | 0.062 |
|  | TMI | 0.178 (0.087) | 0.064 | 0.198 | 0.194 (0.085) | 0.063 | 0.212 | 0.185 (0.090) | 0.066 | 0.206 | 0.203 (0.087) | 0.065 | 0.221 |
|  | MI | 0.002 (0.037) | 0.049 | 0.037 | 0.038 (0.037) | 0.048 | 0.053 | 0.002 (0.038) | 0.050 | 0.038 | 0.045 (0.038) | 0.048 | 0.059 |
|  | MIMP | 0.002 (0.037) | 0.049 | 0.037 | 0.038 (0.037) | 0.048 | 0.053 | 0.002 (0.038) | 0.050 | 0.038 | 0.045 (0.038) | 0.049 | 0.059 |
|  | GBM | 0.099 (0.039) | 0.046 | 0.106 | - | - | - | - | - | - | - | - | - |
|  | GBM + SI + pe | 0.035 (0.038) | 0.047 | 0.052 | - | - | - | - | - | - | - | - | - |
| **MAR1** | SI + pe + pu | 0.002 (0.041) | 0.048 | 0.041 | 0.038 (0.039) | 0.047 | 0.054 | 0.002 (0.042) | 0.049 | 0.042 | 0.044 (0.040) | 0.048 | 0.059 |
|  | SI + pe | 0.002 (0.039) | 0.048 | 0.039 | 0.038 (0.038) | 0.047 | 0.054 | 0.003 (0.040) | 0.049 | 0.040 | 0.044 (0.038) | 0.048 | 0.058 |
|  | TMI | 0.198 (0.076) | 0.056 | 0.212 | 0.216 (0.075) | 0.055 | 0.229 | 0.210 (0.077) | 0.057 | 0.224 | 0.226 (0.076) | 0.056 | 0.238 |
|  | MI | 0.002 (0.035) | 0.049 | 0.035 | 0.038 (0.034) | 0.047 | 0.051 | 0.002 (0.036) | 0.050 | 0.036 | 0.044 (0.034) | 0.048 | 0.056 |
|  | MIMP | 0.001 (0.035) | 0.049 | 0.035 | 0.038 (0.034) | 0.048 | 0.051 | 0.001 (0.036) | 0.050 | 0.036 | 0.044 (0.035) | 0.048 | 0.056 |
|  | GBM | 0.097 (0.035) | 0.046 | 0.103 | - | - | - | - | - | - | - | - | - |
|  | GBM + SI + pe | 0.036 (0.035) | 0.047 | 0.050 | - | - | - | - | - | - | - | - | - |
| **MAR2** | SI + pe + pu | 0.007 (0.044) | 0.048 | 0.045 | 0.041 (0.042) | 0.047 | 0.059 | 0.007 (0.046) | 0.049 | 0.047 | 0.047 (0.043) | 0.048 | 0.064 |
|  | SI + pe | 0.006 (0.041) | 0.048 | 0.041 | 0.041 (0.040) | 0.047 | 0.057 | 0.006 (0.042) | 0.049 | 0.042 | 0.047 (0.040) | 0.048 | 0.062 |
|  | TMI | 0.182 (0.096) | 0.071 | 0.206 | 0.194 (0.094) | 0.069 | 0.216 | 0.194 (0.096) | 0.072 | 0.216 | 0.205 (0.094) | 0.070 | 0.226 |
|  | MI | 0.007 (0.037) | 0.049 | 0.038 | 0.041 (0.036) | 0.048 | 0.055 | 0.007 (0.038) | 0.050 | 0.039 | 0.047 (0.036) | 0.048 | 0.059 |
|  | MIMP | 0.013 (0.037) | 0.050 | 0.039 | 0.049 (0.036) | 0.048 | 0.061 | 0.010 (0.038) | 0.051 | 0.039 | 0.051 (0.036) | 0.049 | 0.062 |
|  | GBM | 0.114 (0.039) | 0.047 | 0.120 | - | - | - | - | - | - | - | - | - |
|  | GBM + SI + pe | 0.044 (0.036) | 0.047 | 0.057 | - | - | - | - | - | - | - | - | - |
| **MAR Sinister** | SI + pe + pu | -0.017 (0.041) | 0.048 | 0.044 | 0.020 (0.040) | 0.047 | 0.045 | -0.020 (0.043) | 0.049 | 0.047 | 0.024 (0.041) | 0.048 | 0.048 |
|  | SI + pe | -0.002 (0.041) | 0.048 | 0.041 | 0.036 (0.040) | 0.047 | 0.054 | -0.002 (0.042) | 0.049 | 0.042 | 0.042 (0.041) | 0.048 | 0.059 |
|  | TMI | 0.203 (0.079) | 0.061 | 0.218 | 0.220 (0.078) | 0.060 | 0.233 | 0.210 (0.082) | 0.063 | 0.225 | 0.228 (0.080) | 0.062 | 0.242 |
|  | MI | 0.000 (0.036) | 0.049 | 0.036 | 0.038 (0.036) | 0.048 | 0.052 | 0.000 (0.037) | 0.050 | 0.037 | 0.045 (0.036) | 0.048 | 0.058 |
|  | MIMP | 0.000 (0.037) | 0.049 | 0.037 | 0.039 (0.036) | 0.048 | 0.053 | 0.000 (0.038) | 0.050 | 0.038 | 0.045 (0.036) | 0.049 | 0.058 |
|  | GBM | 0.106 (0.038) | 0.046 | 0.113 | - | - | - | - | - | - | - | - | - |
|  | GBM + SI + pe | 0.034 (0.037) | 0.047 | 0.050 | - | - | - | - | - | - | - | - | - |

## Table 6. Simulation results for scenario G, n = 1000, 50%missing.

| **Missingness Mechanism** | **Method** | **True confounders** | | | **Leave x1 out** | | | **Add x5** | | | **Leave x1 out + Add x5** | | |
| --- | --- | --- | --- | --- | --- | --- | --- | --- | --- | --- | --- | --- | --- |
|  |  | Bias (SD) | SE | RMSE | Bias (SD) | SE | RMSE | Bias (SD) | SE | RMSE | Bias (SD) | SE | RMSE |
|  | complete | -0.012 (0.029) | 0.050 | 0.031 | 0.040 (0.029) | 0.048 | 0.049 | -0.014 (0.030) | 0.051 | 0.033 | 0.043 (0.029) | 0.048 | 0.052 |
|  | comGBM | 0.027 (0.030) | 0.048 | 0.040 | - | - | - | - | - | - | - | - | - |
| **MCAR** | SI + pe + pu | -0.004 (0.045) | 0.049 | 0.045 | 0.045 (0.042) | 0.047 | 0.062 | -0.005 (0.046) | 0.050 | 0.046 | 0.049 (0.042) | 0.048 | 0.065 |
|  | SI + pe | -0.006 (0.044) | 0.050 | 0.044 | 0.044 (0.042) | 0.048 | 0.061 | -0.008 (0.045) | 0.051 | 0.046 | 0.048 (0.042) | 0.048 | 0.064 |
|  | TMI | 0.174 (0.080) | 0.067 | 0.192 | 0.195 (0.078) | 0.066 | 0.210 | 0.176 (0.080) | 0.069 | 0.193 | 0.199 (0.078) | 0.067 | 0.214 |
|  | MI | -0.005 (0.038) | 0.050 | 0.038 | 0.044 (0.036) | 0.048 | 0.057 | -0.006 (0.039) | 0.051 | 0.039 | 0.048 (0.036) | 0.048 | 0.060 |
|  | MIMP | -0.005 (0.038) | 0.051 | 0.038 | 0.044 (0.037) | 0.048 | 0.057 | -0.006 (0.039) | 0.052 | 0.039 | 0.048 (0.037) | 0.049 | 0.061 |
|  | GBM | 0.085 (0.039) | 0.047 | 0.094 | - | - | - | - | - | - | - | - | - |
|  | GBM + SI + pe | 0.032 (0.038) | 0.047 | 0.050 | - | - | - | - | - | - | - | - | - |
| **MAR1** | SI + pe + pu | 0.024 (0.043) | 0.049 | 0.049 | 0.072 (0.040) | 0.047 | 0.082 | 0.024 (0.045) | 0.049 | 0.051 | 0.076 (0.041) | 0.047 | 0.086 |
|  | SI + pe | 0.026 (0.041) | 0.049 | 0.049 | 0.072 (0.039) | 0.047 | 0.082 | 0.026 (0.042) | 0.049 | 0.049 | 0.076 (0.039) | 0.047 | 0.085 |
|  | TMI | 0.259 (0.057) | 0.054 | 0.265 | 0.281 (0.055) | 0.053 | 0.286 | 0.266 (0.057) | 0.055 | 0.272 | 0.286 (0.056) | 0.054 | 0.291 |
|  | MI | 0.024 (0.036) | 0.049 | 0.043 | 0.071 (0.034) | 0.047 | 0.079 | 0.023 (0.037) | 0.050 | 0.044 | 0.075 (0.035) | 0.048 | 0.083 |
|  | MIMP | 0.023 (0.035) | 0.050 | 0.042 | 0.072 (0.034) | 0.048 | 0.080 | 0.023 (0.036) | 0.051 | 0.043 | 0.075 (0.034) | 0.048 | 0.082 |
|  | GBM | 0.099 (0.036) | 0.047 | 0.105 | - | - | - | - | - | - | - | - | - |
|  | GBM + SI + pe | 0.054 (0.036) | 0.047 | 0.065 | - | - | - | - | - | - | - | - | - |
| **MAR2** | SI + pe + pu | 0.002 (0.046) | 0.049 | 0.046 | 0.048 (0.044) | 0.048 | 0.065 | 0.001 (0.047) | 0.050 | 0.047 | 0.052 (0.044) | 0.048 | 0.068 |
|  | SI + pe | 0.002 (0.044) | 0.049 | 0.044 | 0.049 (0.041) | 0.048 | 0.064 | 0.001 (0.045) | 0.050 | 0.045 | 0.052 (0.042) | 0.048 | 0.067 |
|  | TMI | 0.153 (0.106) | 0.079 | 0.186 | 0.171 (0.098) | 0.076 | 0.197 | 0.157 (0.108) | 0.081 | 0.191 | 0.174 (0.099) | 0.077 | 0.200 |
|  | MI | 0.003 (0.039) | 0.050 | 0.039 | 0.049 (0.037) | 0.048 | 0.061 | 0.001 (0.039) | 0.051 | 0.039 | 0.052 (0.037) | 0.049 | 0.064 |
|  | MIMP | 0.008 (0.039) | 0.051 | 0.040 | 0.055 (0.037) | 0.049 | 0.066 | 0.004 (0.039) | 0.052 | 0.039 | 0.056 (0.037) | 0.049 | 0.067 |
|  | GBM | 0.095 (0.040) | 0.047 | 0.103 | - | - | - | - | - | - | - | - | - |
|  | GBM + SI + pe | 0.041 (0.038) | 0.047 | 0.056 | - | - | - | - | - | - | - | - | - |
| **MAR Sinister** | SI + pe + pu | -0.024 (0.043) | 0.049 | 0.049 | 0.026 (0.040) | 0.047 | 0.048 | -0.028 (0.044) | 0.051 | 0.052 | 0.028 (0.041) | 0.048 | 0.050 |
|  | SI + pe | -0.006 (0.044) | 0.050 | 0.044 | 0.044 (0.041) | 0.048 | 0.060 | -0.007 (0.045) | 0.050 | 0.046 | 0.047 (0.042) | 0.048 | 0.063 |
|  | TMI | 0.205 (0.073) | 0.065 | 0.218 | 0.226 (0.069) | 0.062 | 0.236 | 0.207 (0.076) | 0.066 | 0.221 | 0.230 (0.071) | 0.064 | 0.241 |
|  | MI | -0.006 (0.039) | 0.050 | 0.039 | 0.046 (0.037) | 0.047 | 0.059 | -0.007 (0.039) | 0.051 | 0.040 | 0.049 (0.037) | 0.048 | 0.061 |
|  | MIMP | -0.006 (0.039) | 0.050 | 0.039 | 0.046 (0.037) | 0.048 | 0.059 | -0.007 (0.040) | 0.051 | 0.041 | 0.050 (0.037) | 0.048 | 0.062 |
|  | GBM | 0.094 (0.039) | 0.047 | 0.102 | - | - | - | - | - | - | - | - | - |
|  | GBM + SI + pe | 0.032 (0.038) | 0.047 | 0.050 | - | - | - | - | - | - | - | - | - |

## Table 7. Simulation results for scenario A, n = 5000, 25%missing.

| **Missingness Mechanism** | **Method** | **True confounders** | | | **Leave x1 out** | | | **Add x5** | | | **Leave x1 out + Add x5** | | |
| --- | --- | --- | --- | --- | --- | --- | --- | --- | --- | --- | --- | --- | --- |
|  |  | Bias (SD) | SE | RMSE | Bias (SD) | SE | RMSE | Bias (SD) | SE | RMSE | Bias (SD) | SE | RMSE |
|  | complete | 0.000 (0.013) | 0.021 | 0.013 | 0.038 (0.013) | 0.021 | 0.040 | 0.000 (0.014) | 0.022 | 0.014 | 0.044 (0.014) | 0.021 | 0.046 |
|  | comGBM | 0.019 (0.013) | 0.021 | 0.023 | - | - | - | - | - | - | - | - | - |
| **MCAR** | SI + pe + pu | 0.000 (0.015) | 0.021 | 0.015 | 0.038 (0.015) | 0.021 | 0.041 | 0.000 (0.016) | 0.022 | 0.016 | 0.044 (0.016) | 0.021 | 0.047 |
|  | SI + pe | 0.000 (0.015) | 0.021 | 0.015 | 0.038 (0.015) | 0.021 | 0.041 | 0.000 (0.016) | 0.022 | 0.016 | 0.044 (0.016) | 0.021 | 0.047 |
|  | TMI | 0.115 (0.017) | 0.023 | 0.116 | 0.141 (0.018) | 0.022 | 0.142 | 0.119 (0.018) | 0.023 | 0.120 | 0.148 (0.018) | 0.023 | 0.149 |
|  | MI | 0.000 (0.014) | 0.021 | 0.014 | 0.038 (0.014) | 0.021 | 0.040 | 0.000 (0.015) | 0.022 | 0.015 | 0.044 (0.015) | 0.021 | 0.046 |
|  | MIMP | 0.000 (0.014) | 0.021 | 0.014 | 0.038 (0.014) | 0.021 | 0.040 | 0.000 (0.015) | 0.022 | 0.015 | 0.044 (0.015) | 0.021 | 0.046 |
|  | GBM | 0.058 (0.015) | 0.021 | 0.060 | - | - | - | - | - | - | - | - | - |
|  | GBM + SI + pe | 0.024 (0.015) | 0.021 | 0.028 | - | - | - | - | - | - | - | - | - |
| **MAR1** | SI + pe + pu | 0.000 (0.015) | 0.021 | 0.015 | 0.038 (0.015) | 0.021 | 0.041 | 0.000 (0.016) | 0.022 | 0.016 | 0.044 (0.016) | 0.021 | 0.047 |
|  | SI + pe | 0.000 (0.015) | 0.021 | 0.015 | 0.038 (0.015) | 0.021 | 0.041 | 0.000 (0.016) | 0.022 | 0.016 | 0.044 (0.015) | 0.021 | 0.046 |
|  | TMI | 0.111 (0.017) | 0.022 | 0.112 | 0.139 (0.017) | 0.022 | 0.140 | 0.118 (0.017) | 0.022 | 0.119 | 0.146 (0.017) | 0.022 | 0.147 |
|  | MI | 0.000 (0.014) | 0.021 | 0.014 | 0.038 (0.014) | 0.021 | 0.040 | 0.000 (0.015) | 0.022 | 0.015 | 0.044 (0.015) | 0.021 | 0.046 |
|  | MIMP | 0.000 (0.014) | 0.022 | 0.014 | 0.038 (0.014) | 0.021 | 0.040 | 0.000 (0.014) | 0.022 | 0.014 | 0.044 (0.014) | 0.021 | 0.046 |
|  | GBM | 0.058 (0.015) | 0.021 | 0.060 | - | - | - | - | - | - | - | - | - |
|  | GBM + SI + pe | 0.025 (0.014) | 0.021 | 0.029 | - | - | - | - | - | - | - | - | - |
| **MAR2** | SI + pe + pu | 0.002 (0.016) | 0.021 | 0.016 | 0.038 (0.015) | 0.021 | 0.041 | 0.002 (0.016) | 0.022 | 0.016 | 0.045 (0.016) | 0.021 | 0.048 |
|  | SI + pe | 0.002 (0.015) | 0.021 | 0.015 | 0.038 (0.015) | 0.021 | 0.041 | 0.001 (0.015) | 0.022 | 0.015 | 0.044 (0.016) | 0.021 | 0.047 |
|  | TMI | 0.113 (0.019) | 0.023 | 0.115 | 0.140 (0.019) | 0.022 | 0.141 | 0.120 (0.019) | 0.024 | 0.121 | 0.147 (0.019) | 0.023 | 0.148 |
|  | MI | 0.001 (0.014) | 0.021 | 0.014 | 0.038 (0.014) | 0.021 | 0.040 | 0.001 (0.015) | 0.022 | 0.015 | 0.044 (0.015) | 0.021 | 0.046 |
|  | MIMP | 0.004 (0.014) | 0.022 | 0.015 | 0.042 (0.014) | 0.021 | 0.044 | 0.003 (0.015) | 0.022 | 0.015 | 0.046 (0.015) | 0.021 | 0.048 |
|  | GBM | 0.060 (0.015) | 0.021 | 0.062 | - | - | - | - | - | - | - | - | - |
|  | GBM + SI + pe | 0.027 (0.015) | 0.021 | 0.031 | - | - | - | - | - | - | - | - | - |
| **MAR Sinister** | SI + pe + pu | 0.000 (0.016) | 0.021 | 0.016 | 0.039 (0.015) | 0.021 | 0.042 | 0.001 (0.016) | 0.022 | 0.016 | 0.046 (0.016) | 0.021 | 0.049 |
|  | SI + pe | 0.000 (0.015) | 0.021 | 0.015 | 0.038 (0.015) | 0.021 | 0.041 | 0.000 (0.016) | 0.022 | 0.016 | 0.044 (0.016) | 0.021 | 0.047 |
|  | TMI | 0.120 (0.018) | 0.022 | 0.121 | 0.146 (0.018) | 0.022 | 0.147 | 0.124 (0.018) | 0.023 | 0.125 | 0.153 (0.018) | 0.022 | 0.154 |
|  | MI | 0.000 (0.014) | 0.021 | 0.014 | 0.038 (0.014) | 0.021 | 0.040 | 0.000 (0.015) | 0.022 | 0.015 | 0.045 (0.015) | 0.021 | 0.047 |
|  | MIMP | 0.000 (0.014) | 0.021 | 0.014 | 0.038 (0.014) | 0.021 | 0.040 | 0.000 (0.015) | 0.022 | 0.015 | 0.045 (0.015) | 0.021 | 0.047 |
|  | GBM | 0.061 (0.015) | 0.021 | 0.063 | - | - | - | - | - | - | - | - | - |
|  | GBM + SI + pe | 0.024 (0.015) | 0.021 | 0.028 | - | - | - | - | - | - | - | - | - |

## Table 8. Simulation results for scenario G, n = 5000, 25%missing.

| **Missingness Mechanism** | **Method** | **True confounders** | | | **Leave x1 out** | | | **Add x5** | | | **Leave x1 out + Add x5** | | |
| --- | --- | --- | --- | --- | --- | --- | --- | --- | --- | --- | --- | --- | --- |
|  |  | Bias (SD) | SE | RMSE | Bias (SD) | SE | RMSE | Bias (SD) | SE | RMSE | Bias (SD) | SE | RMSE |
|  | complete | -0.012 (0.014) | 0.022 | 0.018 | 0.040 (0.014) | 0.021 | 0.042 | -0.014 (0.014) | 0.023 | 0.020 | 0.043 (0.014) | 0.022 | 0.045 |
|  | comGBM | 0.021 (0.014) | 0.021 | 0.025 | - | - | - | - | - | - | - | - | - |
| **MCAR** | SI + pe + pu | -0.009 (0.016) | 0.022 | 0.018 | 0.042 (0.016) | 0.021 | 0.045 | -0.010 (0.017) | 0.023 | 0.020 | 0.045 (0.016) | 0.021 | 0.048 |
|  | SI + pe | -0.009 (0.016) | 0.022 | 0.018 | 0.042 (0.016) | 0.021 | 0.045 | -0.010 (0.017) | 0.023 | 0.020 | 0.046 (0.016) | 0.021 | 0.049 |
|  | TMI | 0.102 (0.019) | 0.024 | 0.104 | 0.137 (0.018) | 0.023 | 0.138 | 0.102 (0.020) | 0.025 | 0.104 | 0.140 (0.019) | 0.023 | 0.141 |
|  | MI | -0.009 (0.015) | 0.022 | 0.017 | 0.042 (0.015) | 0.021 | 0.045 | -0.010 (0.016) | 0.023 | 0.019 | 0.045 (0.015) | 0.021 | 0.047 |
|  | MIMP | -0.009 (0.015) | 0.022 | 0.017 | 0.042 (0.015) | 0.021 | 0.045 | -0.011 (0.016) | 0.023 | 0.019 | 0.045 (0.015) | 0.021 | 0.047 |
|  | GBM | 0.052 (0.016) | 0.021 | 0.054 | - | - | - | - | - | - | - | - | - |
|  | GBM + SI + pe | 0.023 (0.015) | 0.021 | 0.027 | - | - | - | - | - | - | - | - | - |
| **MAR1** | SI + pe + pu | 0.000 (0.016) | 0.022 | 0.016 | 0.051 (0.016) | 0.021 | 0.053 | -0.001 (0.016) | 0.022 | 0.016 | 0.054 (0.016) | 0.021 | 0.056 |
|  | SI + pe | 0.001 (0.016) | 0.022 | 0.016 | 0.051 (0.015) | 0.021 | 0.053 | 0.000 (0.016) | 0.022 | 0.016 | 0.055 (0.016) | 0.021 | 0.057 |
|  | TMI | 0.118 (0.018) | 0.023 | 0.119 | 0.154 (0.018) | 0.022 | 0.155 | 0.121 (0.018) | 0.023 | 0.122 | 0.158 (0.018) | 0.022 | 0.159 |
|  | MI | 0.001 (0.015) | 0.022 | 0.015 | 0.051 (0.015) | 0.021 | 0.053 | -0.001 (0.015) | 0.022 | 0.015 | 0.054 (0.015) | 0.021 | 0.056 |
|  | MIMP | 0.000 (0.015) | 0.022 | 0.015 | 0.051 (0.014) | 0.021 | 0.053 | -0.001 (0.015) | 0.023 | 0.015 | 0.054 (0.015) | 0.021 | 0.056 |
|  | GBM | 0.060 (0.015) | 0.021 | 0.062 | - | - | - | - | - | - | - | - | - |
|  | GBM + SI + pe | 0.030 (0.015) | 0.021 | 0.034 | - | - | - | - | - | - | - | - | - |
| **MAR2** | SI + pe + pu | -0.007 (0.016) | 0.022 | 0.017 | 0.043 (0.016) | 0.021 | 0.046 | -0.009 (0.017) | 0.023 | 0.019 | 0.046 (0.016) | 0.021 | 0.049 |
|  | SI + pe | -0.006 (0.016) | 0.022 | 0.017 | 0.043 (0.015) | 0.021 | 0.046 | -0.008 (0.016) | 0.023 | 0.018 | 0.047 (0.015) | 0.021 | 0.049 |
|  | TMI | 0.096 (0.021) | 0.025 | 0.098 | 0.131 (0.020) | 0.023 | 0.133 | 0.098 (0.021) | 0.025 | 0.100 | 0.134 (0.020) | 0.024 | 0.135 |
|  | MI | -0.006 (0.015) | 0.022 | 0.016 | 0.043 (0.015) | 0.021 | 0.046 | -0.008 (0.015) | 0.023 | 0.017 | 0.047 (0.015) | 0.021 | 0.049 |
|  | MIMP | -0.005 (0.015) | 0.022 | 0.016 | 0.046 (0.015) | 0.021 | 0.048 | -0.007 (0.015) | 0.023 | 0.017 | 0.048 (0.015) | 0.022 | 0.050 |
|  | GBM | 0.052 (0.016) | 0.021 | 0.054 | - | - | - | - | - | - | - | - | - |
|  | GBM + SI + pe | 0.025 (0.015) | 0.021 | 0.029 | - | - | - | - | - | - | - | - | - |
| **MAR Sinister** | SI + pe + pu | -0.009 (0.016) | 0.022 | 0.018 | 0.043 (0.015) | 0.021 | 0.046 | -0.010 (0.016) | 0.023 | 0.019 | 0.047 (0.016) | 0.021 | 0.050 |
|  | SI + pe | -0.010 (0.016) | 0.022 | 0.019 | 0.042 (0.016) | 0.021 | 0.045 | -0.011 (0.017) | 0.023 | 0.020 | 0.046 (0.016) | 0.021 | 0.049 |
|  | TMI | 0.106 (0.019) | 0.024 | 0.108 | 0.142 (0.018) | 0.023 | 0.143 | 0.107 (0.020) | 0.024 | 0.109 | 0.145 (0.019) | 0.023 | 0.146 |
|  | MI | -0.009 (0.015) | 0.022 | 0.017 | 0.042 (0.015) | 0.021 | 0.045 | -0.011 (0.015) | 0.023 | 0.019 | 0.046 (0.015) | 0.021 | 0.048 |
|  | MIMP | -0.009 (0.015) | 0.022 | 0.017 | 0.042 (0.015) | 0.021 | 0.045 | -0.011 (0.015) | 0.023 | 0.019 | 0.046 (0.015) | 0.021 | 0.048 |
|  | GBM | 0.054 (0.016) | 0.021 | 0.056 | - | - | - | - | - | - | - | - | - |
|  | GBM + SI + pe | 0.022 (0.016) | 0.021 | 0.027 | - | - | - | - | - | - | - | - | - |

## Table 9. Simulation results for scenario A, n = 5000, 50%missing.

| **Missingness Mechanism** | **Method** | **True confounders** | | | **Leave x1 out** | | | **Add x5** | | | **Leave x1 out + Add x5** | | |
| --- | --- | --- | --- | --- | --- | --- | --- | --- | --- | --- | --- | --- | --- |
|  |  | Bias (SD) | SE | RMSE | Bias (SD) | SE | RMSE | Bias (SD) | SE | RMSE | Bias (SD) | SE | RMSE |
|  | complete | 0.000 (0.013) | 0.021 | 0.013 | 0.038 (0.013) | 0.021 | 0.040 | 0.000 (0.014) | 0.022 | 0.014 | 0.044 (0.014) | 0.021 | 0.046 |
|  | comGBM | 0.019 (0.013) | 0.021 | 0.023 | - | - | - | - | - | - | - | - | - |
| **MCAR** | SI + pe + pu | 0.001 (0.020) | 0.021 | 0.020 | 0.039 (0.019) | 0.021 | 0.043 | 0.002 (0.020) | 0.022 | 0.020 | 0.045 (0.019) | 0.021 | 0.049 |
|  | SI + pe | 0.001 (0.019) | 0.021 | 0.019 | 0.038 (0.018) | 0.021 | 0.042 | 0.002 (0.020) | 0.022 | 0.020 | 0.045 (0.019) | 0.021 | 0.049 |
|  | TMI | 0.189 (0.032) | 0.029 | 0.192 | 0.205 (0.031) | 0.029 | 0.207 | 0.196 (0.032) | 0.030 | 0.199 | 0.213 (0.032) | 0.029 | 0.215 |
|  | MI | 0.002 (0.017) | 0.022 | 0.017 | 0.039 (0.016) | 0.021 | 0.042 | 0.002 (0.017) | 0.022 | 0.017 | 0.045 (0.017) | 0.021 | 0.048 |
|  | MIMP | 0.001 (0.017) | 0.022 | 0.017 | 0.038 (0.016) | 0.021 | 0.041 | 0.002 (0.017) | 0.022 | 0.017 | 0.045 (0.017) | 0.021 | 0.048 |
|  | GBM | 0.094 (0.017) | 0.021 | 0.096 | - | - | - | - | - | - | - | - | - |
|  | GBM + SI + pe | 0.027 (0.018) | 0.021 | 0.032 | - | - | - | - | - | - | - | - | - |
| **MAR1** | SI + pe + pu | 0.000 (0.019) | 0.021 | 0.019 | 0.037 (0.018) | 0.021 | 0.041 | 0.000 (0.020) | 0.022 | 0.020 | 0.043 (0.019) | 0.021 | 0.047 |
|  | SI + pe | 0.002 (0.018) | 0.021 | 0.018 | 0.037 (0.018) | 0.021 | 0.041 | 0.002 (0.019) | 0.022 | 0.019 | 0.043 (0.018) | 0.021 | 0.047 |
|  | TMI | 0.218 (0.024) | 0.025 | 0.219 | 0.236 (0.023) | 0.024 | 0.237 | 0.228 (0.024) | 0.025 | 0.229 | 0.244 (0.024) | 0.025 | 0.245 |
|  | MI | 0.000 (0.016) | 0.022 | 0.016 | 0.037 (0.016) | 0.021 | 0.040 | 0.000 (0.017) | 0.022 | 0.017 | 0.044 (0.016) | 0.021 | 0.047 |
|  | MIMP | 0.000 (0.016) | 0.022 | 0.016 | 0.038 (0.016) | 0.021 | 0.041 | 0.000 (0.016) | 0.022 | 0.016 | 0.044 (0.016) | 0.021 | 0.047 |
|  | GBM | 0.093 (0.016) | 0.021 | 0.094 | - | - | - | - | - | - | - | - | - |
|  | GBM + SI + pe | 0.027 (0.017) | 0.021 | 0.032 | - | - | - | - | - | - | - | - | - |
| **MAR2** | SI + pe + pu | 0.006 (0.020) | 0.021 | 0.021 | 0.040 (0.020) | 0.021 | 0.045 | 0.006 (0.021) | 0.022 | 0.022 | 0.046 (0.021) | 0.021 | 0.051 |
|  | SI + pe | 0.006 (0.019) | 0.021 | 0.020 | 0.040 (0.019) | 0.021 | 0.044 | 0.006 (0.020) | 0.022 | 0.021 | 0.046 (0.019) | 0.021 | 0.050 |
|  | TMI | 0.193 (0.036) | 0.033 | 0.196 | 0.205 (0.034) | 0.032 | 0.208 | 0.203 (0.037) | 0.034 | 0.206 | 0.214 (0.035) | 0.033 | 0.217 |
|  | MI | 0.006 (0.017) | 0.022 | 0.018 | 0.040 (0.017) | 0.021 | 0.043 | 0.006 (0.018) | 0.022 | 0.019 | 0.046 (0.018) | 0.021 | 0.049 |
|  | MIMP | 0.015 (0.017) | 0.022 | 0.023 | 0.051 (0.017) | 0.021 | 0.054 | 0.011 (0.017) | 0.022 | 0.020 | 0.051 (0.017) | 0.022 | 0.054 |
|  | GBM | 0.109 (0.018) | 0.021 | 0.110 | - | - | - | - | - | - | - | - | - |
|  | GBM + SI + pe | 0.035 (0.018) | 0.021 | 0.039 | - | - | - | - | - | - | - | - | - |
| **MAR Sinister** | SI + pe + pu | -0.010 (0.018) | 0.021 | 0.021 | 0.027 (0.018) | 0.021 | 0.032 | -0.010 (0.019) | 0.022 | 0.021 | 0.034 (0.018) | 0.021 | 0.038 |
|  | SI + pe | 0.002 (0.019) | 0.021 | 0.019 | 0.039 (0.018) | 0.021 | 0.043 | 0.002 (0.019) | 0.022 | 0.019 | 0.045 (0.018) | 0.021 | 0.048 |
|  | TMI | 0.202 (0.029) | 0.028 | 0.204 | 0.218 (0.029) | 0.028 | 0.220 | 0.208 (0.030) | 0.029 | 0.210 | 0.226 (0.030) | 0.029 | 0.228 |
|  | MI | -0.002 (0.016) | 0.022 | 0.016 | 0.036 (0.016) | 0.021 | 0.039 | -0.001 (0.017) | 0.022 | 0.017 | 0.042 (0.016) | 0.021 | 0.045 |
|  | MIMP | -0.002 (0.016) | 0.022 | 0.016 | 0.036 (0.016) | 0.021 | 0.039 | -0.001 (0.017) | 0.022 | 0.017 | 0.042 (0.017) | 0.021 | 0.045 |
|  | GBM | 0.098 (0.017) | 0.021 | 0.099 | - | - | - | - | - | - | - | - | - |
|  | GBM + SI + pe | 0.027 (0.017) | 0.021 | 0.032 | - | - | - | - | - | - | - | - | - |

## Table 10. Simulation results for scenario G, n = 5000, 50%missing.

| **Missingness Mechanism** | **Method** | **True confounders** | | | **Leave x1 out** | | | **Add x5** | | | **Leave x1 out + Add x5** | | |
| --- | --- | --- | --- | --- | --- | --- | --- | --- | --- | --- | --- | --- | --- |
|  |  | Bias (SD) | SE | RMSE | Bias (SD) | SE | RMSE | Bias (SD) | SE | RMSE | Bias (SD) | SE | RMSE |
|  | complete | -0.012 (0.014) | 0.022 | 0.018 | 0.040 (0.014) | 0.021 | 0.042 | -0.014 (0.014) | 0.023 | 0.020 | 0.043 (0.014) | 0.022 | 0.045 |
|  | comGBM | 0.021 (0.014) | 0.021 | 0.025 | - | - | - | - | - | - | - | - | - |
| **MCAR** | SI + pe + pu | -0.006 (0.021) | 0.022 | 0.022 | 0.044 (0.019) | 0.021 | 0.048 | -0.007 (0.021) | 0.022 | 0.022 | 0.048 (0.020) | 0.021 | 0.052 |
|  | SI + pe | -0.005 (0.020) | 0.022 | 0.021 | 0.045 (0.018) | 0.021 | 0.048 | -0.005 (0.020) | 0.022 | 0.021 | 0.048 (0.019) | 0.021 | 0.052 |
|  | TMI | 0.178 (0.035) | 0.032 | 0.181 | 0.199 (0.033) | 0.030 | 0.202 | 0.180 (0.036) | 0.033 | 0.184 | 0.203 (0.033) | 0.031 | 0.206 |
|  | MI | -0.005 (0.017) | 0.022 | 0.018 | 0.044 (0.017) | 0.021 | 0.047 | -0.006 (0.018) | 0.023 | 0.019 | 0.048 (0.017) | 0.021 | 0.051 |
|  | MIMP | -0.006 (0.018) | 0.022 | 0.019 | 0.044 (0.017) | 0.021 | 0.047 | -0.007 (0.018) | 0.023 | 0.019 | 0.048 (0.017) | 0.021 | 0.051 |
|  | GBM | 0.081 (0.017) | 0.021 | 0.083 | - | - | - | - | - | - | - | - | - |
|  | GBM + SI + pe | 0.024 (0.018) | 0.021 | 0.030 | - | - | - | - | - | - | - | - | - |
| **MAR1** | SI + pe + pu | 0.023 (0.020) | 0.022 | 0.030 | 0.071 (0.019) | 0.021 | 0.073 | 0.022 (0.021) | 0.022 | 0.030 | 0.075 (0.019) | 0.021 | 0.077 |
|  | SI + pe | 0.026 (0.019) | 0.022 | 0.032 | 0.071 (0.018) | 0.021 | 0.073 | 0.025 (0.019) | 0.022 | 0.031 | 0.075 (0.018) | 0.021 | 0.077 |
|  | TMI | 0.267 (0.023) | 0.024 | 0.268 | 0.289 (0.022) | 0.023 | 0.290 | 0.273 (0.024) | 0.024 | 0.274 | 0.294 (0.023) | 0.024 | 0.295 |
|  | MI | 0.023 (0.017) | 0.022 | 0.029 | 0.071 (0.017) | 0.021 | 0.073 | 0.022 (0.018) | 0.022 | 0.028 | 0.075 (0.017) | 0.021 | 0.077 |
|  | MIMP | 0.022 (0.017) | 0.022 | 0.028 | 0.071 (0.016) | 0.021 | 0.073 | 0.021 (0.017) | 0.022 | 0.027 | 0.074 (0.016) | 0.021 | 0.076 |
|  | GBM | 0.096 (0.017) | 0.021 | 0.097 | - | - | - | - | - | - | - | - | - |
|  | GBM + SI + pe | 0.047 (0.017) | 0.021 | 0.050 | - | - | - | - | - | - | - | - | - |
| **MAR2** | SI + pe + pu | 0.004 (0.021) | 0.022 | 0.021 | 0.049 (0.020) | 0.021 | 0.053 | 0.002 (0.021) | 0.022 | 0.021 | 0.052 (0.020) | 0.021 | 0.056 |
|  | SI + pe | 0.003 (0.020) | 0.022 | 0.020 | 0.049 (0.019) | 0.021 | 0.053 | 0.002 (0.020) | 0.022 | 0.020 | 0.052 (0.019) | 0.021 | 0.055 |
|  | TMI | 0.161 (0.042) | 0.037 | 0.166 | 0.178 (0.039) | 0.035 | 0.182 | 0.165 (0.043) | 0.038 | 0.171 | 0.181 (0.039) | 0.036 | 0.185 |
|  | MI | 0.004 (0.018) | 0.022 | 0.018 | 0.049 (0.017) | 0.021 | 0.052 | 0.002 (0.018) | 0.022 | 0.018 | 0.053 (0.018) | 0.021 | 0.056 |
|  | MIMP | 0.011 (0.018) | 0.022 | 0.021 | 0.058 (0.017) | 0.021 | 0.060 | 0.007 (0.018) | 0.023 | 0.019 | 0.057 (0.017) | 0.022 | 0.059 |
|  | GBM | 0.091 (0.018) | 0.021 | 0.093 | - | - | - | - | - | - | - | - | - |
|  | GBM + SI + pe | 0.033 (0.018) | 0.021 | 0.038 | - | - | - | - | - | - | - | - | - |
| **MAR Sinister** | SI + pe + pu | -0.018 (0.019) | 0.022 | 0.026 | 0.032 (0.018) | 0.021 | 0.037 | -0.018 (0.020) | 0.022 | 0.027 | 0.036 (0.018) | 0.021 | 0.040 |
|  | SI + pe | -0.005 (0.019) | 0.022 | 0.020 | 0.045 (0.018) | 0.021 | 0.048 | -0.006 (0.020) | 0.022 | 0.021 | 0.049 (0.019) | 0.021 | 0.053 |
|  | TMI | 0.189 (0.033) | 0.031 | 0.192 | 0.210 (0.031) | 0.030 | 0.212 | 0.190 (0.035) | 0.032 | 0.193 | 0.214 (0.032) | 0.031 | 0.216 |
|  | MI | -0.009 (0.017) | 0.022 | 0.019 | 0.041 (0.017) | 0.021 | 0.044 | -0.010 (0.018) | 0.023 | 0.021 | 0.045 (0.017) | 0.021 | 0.048 |
|  | MIMP | -0.009 (0.018) | 0.022 | 0.020 | 0.041 (0.017) | 0.021 | 0.044 | -0.010 (0.018) | 0.023 | 0.021 | 0.045 (0.017) | 0.022 | 0.048 |
|  | GBM | 0.084 (0.017) | 0.021 | 0.086 | - | - | - | - | - | - | - | - | - |
|  | GBM + SI + pe | 0.024 (0.018) | 0.021 | 0.030 | - | - | - | - | - | - | - | - | - |

## Table 11. Simulation results for scenario G with the correct logistic model (50%missing).

| **Missingness Mechanism** | **Method** | **n = 500** | | | **n = 1000** | | | **n = 5000** | | |
| --- | --- | --- | --- | --- | --- | --- | --- | --- | --- | --- |
|  |  | Bias (SD) | SE | RMSE | Bias (SD) | SE | RMSE | Bias (SD) | SE | RMSE |
|  | complete | -0.002 (0.045) | 0.070 | 0.045 | 0.000 (0.030) | 0.049 | 0.030 | 0.001 (0.014) | 0.022 | 0.014 |
| **MCAR** | SI + pe + pu | 0.001 (0.070) | 0.070 | 0.070 | 0.002 (0.045) | 0.049 | 0.045 | 0.001 (0.020) | 0.022 | 0.020 |
|  | SI + pe | -0.001 (0.062) | 0.070 | 0.062 | 0.000 (0.045) | 0.049 | 0.045 | 0.002 (0.020) | 0.022 | 0.020 |
|  | TMI | -0.050 (0.129) | 0.098 | 0.138 | -0.045 (0.078) | 0.067 | 0.090 | -0.037 (0.035) | 0.030 | 0.051 |
|  | MI | 0.001 (0.056) | 0.072 | 0.056 | 0.001 (0.038) | 0.050 | 0.038 | 0.001 (0.017) | 0.022 | 0.017 |
|  | MIMP | 0.001 (0.056) | 0.072 | 0.056 | 0.001 (0.038) | 0.050 | 0.038 | 0.001 (0.017) | 0.022 | 0.017 |
| **MAR1** | SI + pe + pu | 0.027 (0.063) | 0.069 | 0.069 | 0.030 (0.043) | 0.048 | 0.052 | 0.029 (0.020) | 0.021 | 0.035 |
|  | SI + pe | 0.028 (0.059) | 0.069 | 0.065 | 0.032 (0.041) | 0.048 | 0.052 | 0.031 (0.019) | 0.021 | 0.036 |
|  | TMI | 0.065 (0.098) | 0.083 | 0.118 | 0.073 (0.055) | 0.056 | 0.091 | 0.077 (0.025) | 0.024 | 0.081 |
|  | MI | 0.028 (0.053) | 0.070 | 0.060 | 0.030 (0.036) | 0.049 | 0.047 | 0.029 (0.017) | 0.021 | 0.034 |
|  | MIMP | 0.028 (0.053) | 0.071 | 0.060 | 0.029 (0.035) | 0.049 | 0.045 | 0.028 (0.017) | 0.022 | 0.033 |
| **MAR2** | SI + pe + pu | 0.007 (0.067) | 0.070 | 0.067 | 0.008 (0.046) | 0.049 | 0.047 | 0.009 (0.021) | 0.022 | 0.023 |
|  | SI + pe | 0.004 (0.063) | 0.070 | 0.063 | 0.008 (0.044) | 0.049 | 0.045 | 0.009 (0.020) | 0.022 | 0.022 |
|  | TMI | -0.087 (0.167) | 0.110 | 0.188 | -0.085 (0.133) | 0.083 | 0.158 | -0.072 (0.046) | 0.037 | 0.085 |
|  | MI | 0.008 (0.057) | 0.070 | 0.058 | 0.008 (0.039) | 0.050 | 0.040 | 0.009 (0.018) | 0.022 | 0.020 |
|  | MIMP | 0.011 (0.057) | 0.073 | 0.058 | 0.014 (0.039) | 0.051 | 0.041 | 0.017 (0.018) | 0.022 | 0.025 |
| **MAR sinister** | SI + pe + pu | 0.019 (0.061) | 0.069 | 0.064 | -0.017 (0.043) | 0.049 | 0.046 | -0.011 (0.019) | 0.022 | 0.022 |
|  | SI + pe | 0.005 (0.065) | 0.071 | 0.065 | 0.000 (0.043) | 0.049 | 0.043 | 0.002 (0.019) | 0.022 | 0.019 |
|  | TMI | -0.008 (0.112) | 0.091 | 0.112 | -0.013 (0.081) | 0.064 | 0.082 | -0.026 (0.033) | 0.029 | 0.042 |
|  | MI | 0.013 (0.056) | 0.072 | 0.057 | 0.000 (0.038) | 0.050 | 0.038 | -0.003 (0.017) | 0.022 | 0.017 |
|  | MIMP | 0.013 (0.056) | 0.072 | 0.057 | 0.000 (0.039) | 0.050 | 0.039 | -0.003 (0.017) | 0.022 | 0.017 |

*Note.* **Complete**: logistic regression with complete data before introducing missingness**; SI + pe + pu:** single imputation + prediction error + parameter uncertainty; **SI + pe**: single imputation + prediction error; **TMI**: treatment mean imputation; **MI**: multiple imputation (m = 20); **MIMP**: multiple imputation missingness pattern (m = 20); **SD**: standard deviation; **SE**: standard error; **RMSE**: root mean squared error.
